# Plasma miRNA profile at COVID-19 onset predicts severity status and mortality

**DOI:** 10.1080/22221751.2022.2038021

**Published:** 2022-02-27

**Authors:** Asier Fernández-Pato, Ana Virseda-Berdices, Salvador Resino, Pablo Ryan, Oscar Martínez-González, Felipe Pérez-García, María Martin-Vicente, Daniel Valle-Millares, Oscar Brochado-Kith, Rafael Blancas, Amalia Martínez, Francisco C. Ceballos, Sofía Bartolome-Sánchez, Erick Joan Vidal-Alcántara, David Alonso, Natalia Blanca-López, Ignacio Ramirez Martinez-Acitores, Laura Martin-Pedraza, María Ángeles Jiménez-Sousa, Amanda Fernández-Rodríguez

**Affiliations:** a Unit of Viral Infection and Immunity, National Center for Microbiology (CNM), Health Institute Carlos III (ISCIII), Madrid, Spain; b Department of Genetics, University of Groningen, University Medical Center Groningen, Groningen, The Netherlands; c Centro de Investigación Biomédica en Red de Enfermedades Infecciosas, Instituto de Salud Carlos III, Madrid, Spain; d Department of Infectious Diseases, Hospital Universitario Infanta Leonor, Madrid, Spain; e School of Medicine, Complutense University of Madrid, Madrid, Spain; f Gregorio Marañón Health Research Institute, Madrid, Spain; g Critical Care Department, Hospital Universitario del Tajo, Aranjuez, Spain; h Clinical Microbiology Department, Hospital Universitario Príncipe de Asturias, Alcalá de Henares, Spain; i Department of Biomedicine and Biotechnology, Faculty of Medicine, University of Alcalá de Henares, Madrid, Spain; j Internal Medicine Service, Hospital Universitario Príncipe de Asturias, Alcalá de Henares, Spain; k Allergology Department, University Hospital Infanta Leonor, Madrid, Spain

**Keywords:** SARS-CoV2, COVID-19, miRNAs, severity, mortality

## Abstract

**Background:**

MicroRNAs (miRNAs) have a crucial role in regulating immune response against infectious diseases, showing changes early in disease onset and before the detection of the pathogen. Thus, we aimed to analyze the plasma miRNA profile at COVID-19 onset to identify miRNAs as early prognostic biomarkers of severity and survival.

**Methods and results:**

Plasma miRNome of 96 COVID-19 patients that developed asymptomatic/mild, moderate and severe disease was sequenced together with a group of healthy controls. Plasma immune-related biomarkers were also assessed. COVID-19 patients showed 200 significant differentially expressed (SDE) miRNAs concerning healthy controls, with upregulated putative targets of SARS-CoV-2, and inflammatory miRNAs. Among COVID-19 patients, 75 SDE miRNAs were observed in asymptomatic/mild compared to symptomatic patients, which were involved in platelet aggregation and cytokine pathways, among others. Moreover, 137 SDE miRNAs were identified between severe and moderate patients, where miRNAs targeting the SARS CoV-2 genome were the most strongly disrupted. Finally, we constructed a mortality predictive risk score (miRNA-MRS) with ten miRNAs. Patients with higher values had a higher risk of 90-days mortality (hazard ratio = 4.60; *p*-value < 0.001). Besides, the discriminant power of miRNA-MRS was significantly higher than the observed for age and gender (AUROC = 0.970 vs. 0.881; *p* = 0.042).

**Conclusions:**

SARS-CoV-2 infection deeply disturbs the plasma miRNome from an early stage of COVID-19, making miRNAs highly valuable as early predictors of severity and mortality.

## Introduction

The pathogenesis of the severe acute respiratory syndrome coronavirus 2 (SARS-CoV-2) infection and the virus-host interactions during COVID-19 are still not entirely understood. Therefore, it is essential to continue unraveling the biological and molecular mechanisms behind the disparity in disease severity. Omics approaches are indispensable in this scenario, generating crucial information for decision-making in public health policies [1] and allowing the identification of new biomarkers for predicting severity in COVID-19 patients.

MicroRNAs (miRNAs) are small non-coding RNAs that play a fundamental role in regulating gene expression by binding the 3´ untranslated region of target RNAs. This post-transcriptional regulation is essential in both innate and adaptive immunity [2], and the fine-tune of the immune response. Several miRNAs are key regulators of inflammation-related mediators, being essential in some inflammatory diseases [2]. In addition, evidences point to an essential function of miRNAs in the pathogenesis and therapeutics of different viral diseases, including those related to the respiratory tract [3], such as the respiratory syncytial virus (RSV) [4], middle east respiratory syndrome coronavirus (MERS-CoV) [5], SARS-CoV [6], and SARS-CoV-2 [7].

In this regard, several host miRNAs have been identified to target the SARS-CoV-2 genome, repressing its expression and mitigating the pathogenesis of COVID-19. Therefore, the interaction between SARS-CoV-2 and the cellular miRNome could modify the outcome of the infection, as some mutations in the 3´UTR region of the viral genome leads to a viral escape from the host immune system [8]. SARS-CoV-2 infection induces a robust host miRNA response, as it was observed in patients admitted to intensive care units (ICU) [6]. However, it is unknown the miRNA profile of other severity outcomes, specially at disease onset, which could give new insights to improve COVID-19 detection and management.

Therefore, miRNAs could have a potential role in counteracting the SARS-CoV-2 induction of inflammatory response [9]. In this setting, miRNAs are deeply involved in regulating the expression of cytokines, chemokines, and growth factors [10–12], which is directly related with the cytokine release syndrome (CRS) of COVID-19. To date, the plasma miRNome of COVID-19 patients of different severity grades has not been massively assessed. The integrative analysis of the miRNome data with plasma cytokine levels could unravel the miRNA-cytokine interplay in COVID-19, identifying potential miRNAs as new therapeutic approaches against CRS and putative survival biomarkers at disease onset.

This study aimed to characterize the plasma miRNome at the onset of COVID-19 of different severity statuses and to identify a miRNA signature of mortality.

## Methods

### Design and study population

A multicenter observational study was performed in 96 patients with SARS-CoV-2 infection enrolled from March to August 2020 at three public hospitals from Madrid: University Hospital Infanta Leonor, Del Tajo University Hospital, and Príncipe de Asturias University Hospital. The study protocol was approved by the Ethics Committee of the Institute of Health Carlos III (PI 33_2020-v3) and the Ethics Committee of each hospital. Written, oral, or delegated informed consent was obtained from patients or legal representatives whenever possible. The ethics committee, in some cases, authorized an informed consent waiver.

Patients were classified according to their highest severity grade during the evolution of COVID-19 as follows: 1) Severe: i) death during hospitalization, ii) ICU admission, iii) invasive mechanical ventilation, or iv) presence of bilateral pulmonary infiltrates, mechanical ventilation and oxygen saturation (Sat0_2_) ≤ 93%; 2) Moderate: the remaining patients admitted to hospital who did not fulfil severe COVID-19 criteria; 3) Asymptomatic/Mild (AM): individuals with minor or no COVID-19 symptoms; 4) Control: healthy donors recruited before COVID-19 outbreak. The STROBE-ID checklist was used to strengthen the design and conduct the study.

### Clinical data and sample collection

Epidemiological and clinical variables were exhaustively collected from medical records using an electronic case report form (eCRF) built using REDCap electronic data capture tools [13]. Plasma samples were collected at hospital entry or within the first days after hospitalization and before treatment with specific therapies for COVID-19 such as immunotherapy (tocilizumab, interferon-beta (IFNβ), corticoids, or ribavirin, among others).

### Analysis of miRNome by high throughput sequencing

Total RNA, including small RNAs, was isolated from 400μl of plasma with the miRNeasy Serum Plasma Advanced kit (Qiagen, Hilden, Germany) following manufacturer's instructions. RNA quality and quantity were evaluated by the Bioanalyzer 2100 with Agilent RNA 6000 Nano Kit. Small RNA libraries were constructed using the NEBNext Multiplex Small RNA Library Prep for Illumina (New England Biolabs, Ipswich, MA, USA) at Parque Científico of Madrid (Spain). Next, small RNAs were sequenced in NovaSeq 6000, Illumina, at the National Centre for Microbiology (Majadahonda, Spain), estimating over 50 million reads per sample (**Supplementary Data 1**).

Raw data were analyzed using a specific bioinformatic pipeline detailed in **Supplementary Data 2**. Briefly, reads were quality checked with FastQC (v.0.11.3), and adapter sequences were trimmed with cutadapt (v.1.13). Reads were then processed with miRDeep2 (v.0.0.7) to identify and quantify known miRNAs from the reference human genome (GRCh38) and miRBase (v2.0) [14].

### Cytokines and chemokines quantification

Twenty-six plasma markers previously related to COVID-19 (**Supplementary Data 3**) were evaluated with a custom Procartaplex multiplex immunoassay (Invitrogen) using the Bio-plex 200^TM^ system (BioRad Laboratories, Hercules, California, USA) following the manufacturer’s specifications. We used the raw fluorescence intensity (FI) values as a relative quantification of the analyte abundances, as previously described [15].

### Outcome variables

We analyzed several outcomes: i) infection: all SARS-CoV-2 infected patients were compared to healthy controls; ii) Symptomatology: symptomatic patients (moderate plus severe) versus asymptomatic; iii) Severity: severe versus moderate patients; iv) Mortality: 90-days mortality of SARS-CoV-2 infected patients, where the baseline was the date of diagnosis of SARS-CoV-2 infection.

### Statistical analysis

The statistical analysis was performed by R statistical package (v4.0.3). Detailed statistical analysis information is available in **Supplementary Data 4**.

The Kruskal–Wallis test and Pearson’s chi-squared test were used for descriptive data for continuous and discrete variables. miRNA counts were normalized with the DESeq2 method (v1.30.0), and expression differences between severity groups were analyzed using a generalized linear model (GLM) with negative binomial distribution adjusted by age and gender. miRNAs with fold change (FC) ≥ 1.5 and q-value ≤ 0.05 (*p*-value corrected for the false discovery rate (FDR) by Benjamini-Hochberg correction) were considered significant.

Mortality analysis for 90 days after hospitalization was performed by a Cox Proportional-Hazard regression model adjusted by age and gender. Variance stabilizing transformation (VST) normalized counts of all miRNAs were included in the least absolute shrinkage and selection operator (LASSO) multivariate Cox regression model to select the miRNAs with the best predictive value and construct the miRNA mortality risk score (miRNA-MRS) within 90 days of hospital admission (see **Supplementary Data 4** for extended information). The predictive performance for miRNA-MRS was assessed using the area under the receiver-operating characteristic curve (AUROC) and by calculating sensitivity, specificity and positive and negative predictive value.

Plasma cytokine and chemokine FI values were preprocessed with a weighted Box–Cox, followed by quantile normalization [16]. Normalized data were analyzed with a GLM with gamma distribution. Correlation analysis between SDE miRNAs of each contrast and cytokines/chemokines was performed using the Spearman correlation test (q-value < 0.1).

### miRNA-based target prediction and pathway enrichment analysis

Significant differentially expressed (SDE) miRNAs were analyzed for experimentally validated miRNA-target interactions with MIENTURNET (MicroRNA ENrichment TURned NETwork) [17]. Gene targets with at least three validated interactions with SDE miRNAs were selected to perform functional enrichment analysis with the Reactome Pathway database [18]. Enrichment *p*-values (Fischer’s exact test with hypergeometric distribution) were corrected for false discovery ratio (FDR) (q-value ≤ 0.1).

## Results

### Patient characteristics

Characteristics of the four groups of patients are shown in Table 1. There were no differences in the time point at which the plasma samples were obtained among groups, neither between symptoms onset and sampling. Ethnicity, smoker status, arterial hypertension, chronic pulmonary disease, COVID-19-related symptoms, and basal therapy with nonsteroidal anti-inflammatory drugs showed significant differences between groups. COVID-19 hospitalization requirements were found significantly different between groups, as they were used for severity definition (see methods): i.e. oxygenotherapy, mechanical ventilation, presence of bilateral pulmonary infiltrates, ICU and exitus.

**Table 1.** Clinical, epidemiological, and virological characteristics of SARS-CoV-2 infected patients.

	Severe	Moderate	AM	Controls	*p*-value
No.	32	52	12	13	
Age (years)	63.4 (52.9-78.3)	59.4 (53.0- 71.8)	66.2 (44.4- 72.6)	66.7 (57- 68.9)	0.854
Gender (male)	23 / 32 (71.9%)	26 / 52 (50.0%)	4 / 12 (33.3%)	7 / 13 (53.8%)	0.090
Ethnicity (n = 105)					
* Caucasian*	25 / 32 (78.1%)	29 / 49 (59.2%)	12 / 12 (100%)	12 / 12 (100%)	0.002
* Hispanic*	7 / 32 (21.9%)	18 / 49 (36.7%)	0 / 12 (0%)	0 / 12 (0%)	0.007
BMI (kg/m^2^) (n = 49)	28.3 (23.6- 36.3)	29.9 (27.6- 32.8)	28.4 (25.7- 29.3)	28.1 (25.8- 30.4)	0.733
BMI ≥25 (kg/m^2^) (n = 49)	14 / 20 (70%)	11 / 12 (91.7%)	6 / 8 (75%)	7 / 9 (77.8%)	0.560
Smoker status					
* Smoker*	2 / 32 (6.2%)	4 / 52 (7.7%)	2 / 12 (16.7%)	NA	0.522
* Ex-smoker*	7 / 32 (21.9%)	5 / 52 (9.6%)	4 / 12 (33.3%)	NA	0.087
Time between hospitalization to sampling	1.5 (0-5)	2 (0-3.2)	1 (0.5-1.5)	NA	0.740
Time between symptoms and sampling	6 (3-8)	7.5 (4-10.2)	–	NA	0.634
**Comorbidities**					
Arterial hypertension	14 / 32 (43.8%)	23 / 52 (44.2%)	5 / 12 (41.7%)	NA	0.987
Cardiopathy	5 / 32 (15.6%)	10 / 52 (19.2%)	2 / 12 (16.7%)	NA	0.911
Chronic pulmonary disease	9 / 32 (28.1%)	7 / 52 (13.5%)	0 / 12 (0%)	NA	0.055
Chronic renal disease	6 / 32 (18.8%)	4 / 52 (7.7%)	2 / 12 (16.7%)	NA	0.296
Chronic liver disease	1 / 32 (3.1%)	1 / 52 (1.9%)	0 / 12 (0%)	NA	0.806
Chronic neurological disease	7 / 32 (21.9%)	7 / 52 (13.5%)	0 / 12 (0%)	NA	0.177
Neoplasia	3 / 32 (9.4%)	2 / 52 (3.8%)	3 / 12 (25.0%)	NA	0.056
Obesity	9 / 32 (28.1%)	9 / 52 (17.3%)	2 / 12 (16.7%)	NA	0.461
Diabetes	7 / 32 (21.9%)	9 / 52 (17.3%)	4 / 12 (33.3%)	NA	0.461
Chronic inflammatory disease	3 / 32 (9.4%)	1 / 52 (1.9%)	0 / 12 (0%)	NA	0.187
Autoinmune diseases	3 / 32 (9.4%)	1 / 52 (1.9%)	0 / 12 (0%)	NA	0.187
**Therapy**					
***Basal***					
NSAIDs	4 / 32 (12.5%)	0 / 52 (0%)	0 / 12 (0%)	NA	0.015
ACE inhibitors	6 / 32 (18.8%)	10 / 52 (19.2%)	0 / 12 (0%)	NA	0.253
ARA II	3 / 32 (9.4%)	5 / 52 (9.6%)	1 / 12 (8.3%)	NA	0.991
Corticoids	4 / 32 (12.5%)	6 / 52 (11.5%)	0 / 12 (0%)	NA	0.446
HIV antiretroviral therapy	1 / 32 (3.1%)	1 / 52 (1.9%)	0 / 12 (0%)	0 / 13 (0%)	0.858
***Treatment***					
Chloroquine and hidroxychloroquine	23 / 32 (71.9%)	51 / 52 (98.1%)	0 / 12 (0%)	NA	<0.001
Tocilizumab	13 / 32 (40.6%)	10 / 52 (19.2%)	0 / 12 (0%)	NA	0.009
Corticoids	27 / 32 (84.4%)	21 / 52 (40.4%)	0 / 12 (0%)	NA	<0.001
**COVID-19 related symptoms**					
Dyspnoea	28 / 32 (87.5%)	33 / 52 (63.5%)	1 / 12 (8.3%)	NA	<0.001
Cough	22 / 32 (68.8%)	40 / 52 (76.9%)	3 / 12 (25%)	NA	0.002
Headache	7 / 32 (21.9%)	22 / 52 (42.3%)	0 / 12 (0%)	NA	0.007
Diarrhea or abdominal pain	12 / 32 (37.5%)	28 / 52 (53.8%)	2 / 12 (16.7%)	NA	0.044
**Hospitalization**					
Hospital stay (days) (n = 86)	18 (10.7- 25.2)	8.5 (6- 12.2)	9 (8.5- 9.5)	NA	<0.001
Maximum temperature (n = 87)	38.1 (37.5- 38.8)	38 (37.28- 38.5)	36.4 (36.3- 37.7)	NA	0.232
Oxygenotherapy	31 / 32 (96.9%)	30 / 52 (57.7%)	2 / 12 (16.7%)	NA	<0.001
Invasive mechanical ventilation	9 / 32 (28.1%)	0 / 52 (0%)	0 / 12 (0%)	NA	<0.001
Non- Invasive mechanical ventilation	15 / 32 (46.9%)	3 / 52 (5.8%)	0 / 12 (0%)	NA	<0.001
Infiltrates	32 / 32 (100%)	47 / 52 (90.4%)	0 / 12 (0%)	NA	<0.001
ICU	13 / 32 (40.6%)	0 / 52 (0%)	0 / 12 (0%)	NA	<0.001
Exitus	13 / 32 (40.6%)	0 / 52 (0%)	0 / 12 (0%)	NA	<0.001

**Statistics**: The values are expressed as the absolute number (%) and median (interquartile range). *P-values* were estimated by Kruskal-Wallis test for continuous variables and Pearson’s chi-squared test for categorical variables. **Abbreviations**: AM, Asymptomatic/Mild, BMI, body mass index, NSAIDs, nonsteroidal anti-inflammatory drugs; ACE, angiotensin-converting enzyme; ARA, angiotensin receptor antagonists: HIV, human immunodeficiency virus; ICU, intensive care unit; NA, not available./ not applicable

### miRNome characterization and correlation with cytokine release by severity groups

We obtained 50 million reads per sample, roughly three times over the minimum depth required for miRNA expression analysis. A total of 2656 miRNAs (91.97% of miRBase miRNAs) were identified, and 767 miRNAs were successfully filtered with sufficiently large counts for the differential expression analysis.

**Figure 1.**
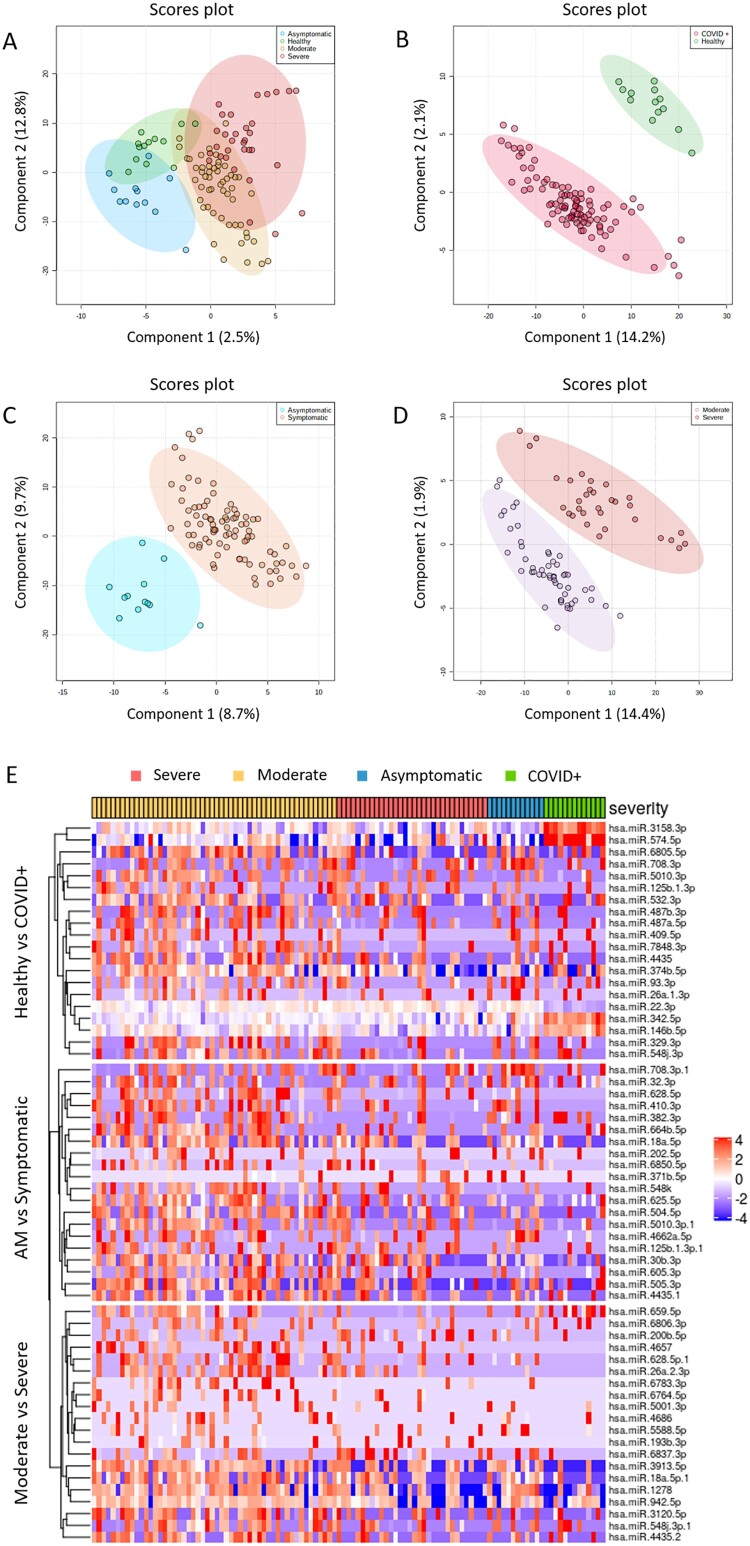
Exploratory analysis. A to D. Multivariate analysis was performed by supervised partial least squares discriminant analysis (PLS-DA) from normalized log transformed and scaled miRNA expression data: A) all individuals (healthy controls, asymptomatic, moderate and severe); B) COVID+ patients vs. healthy controls; C) AM vs symptomatic; D) Severe vs moderate. Each symbol represents the miRNA profile of each participant of the study. E) Heatmap and hierarchical clustering of the SDE miRNAs. Top 20 SDE miRNAs for each comparison are shown: Healthy vs COVID+, AM vs Symptomatic and Moderate vs Severe. Study subjects are represented in columns and SDE miRNAs in rows, with clustering dendograms on the left for miRNAs and at the top for samples. The colour scale shows the relative expression level of SDE miRNAs. Red colour indicates a higher expression level and blue a lower expression level. Patients are grouped by severity. AM, asymptomatic; SDE, significant differentially expressed.

The partial least squares discriminant analysis (PLS-DA) showed differences between severity groups according to the miRNome expression (Figure 1**A-D**), where each group showed a specific expression pattern (Figure 1**E)**.

**Figure 2.**
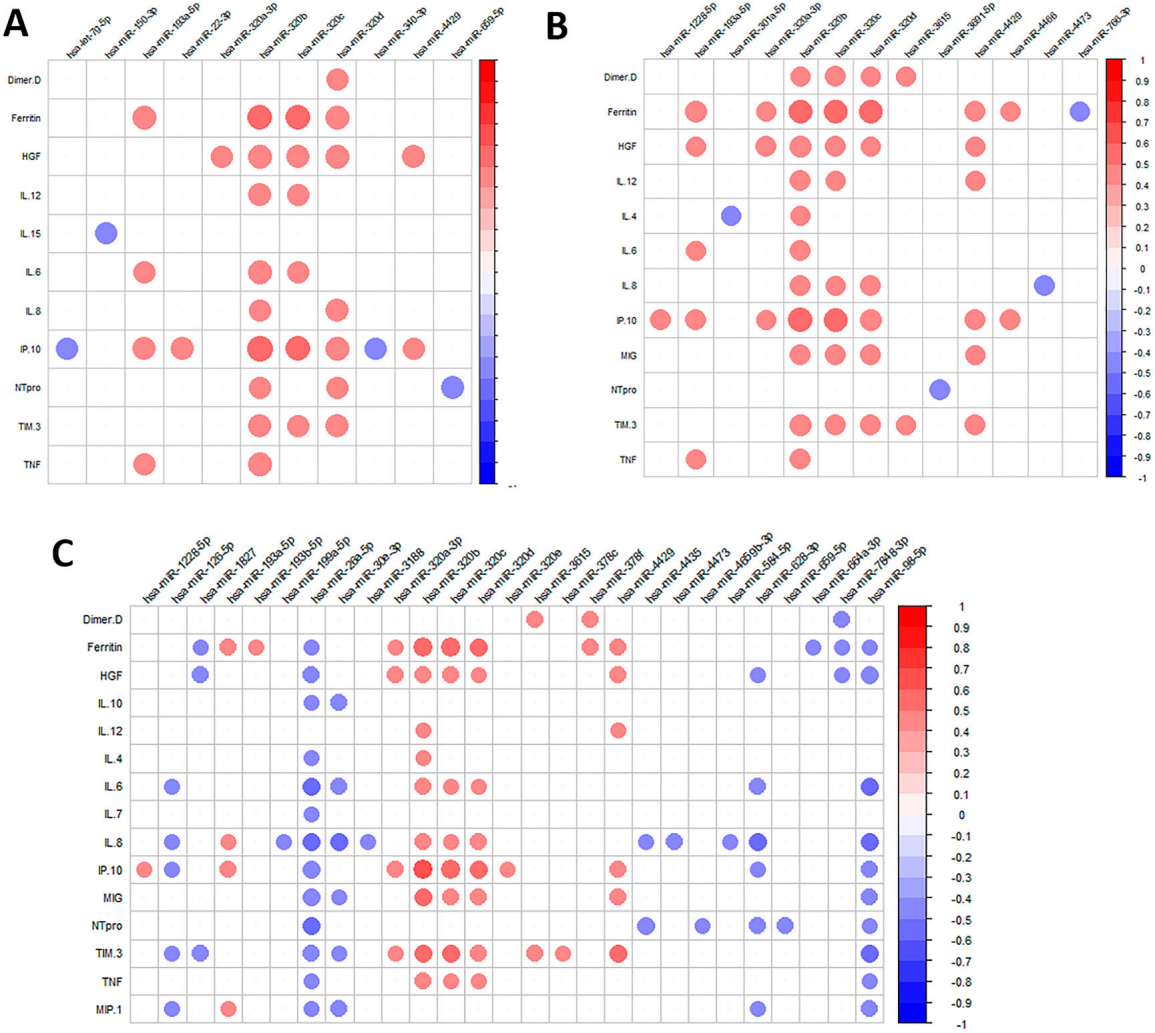
**Pearson correlation plot showing the correlation between SDE miRNAs and plasma cytokines/chemokines considering all patients for each comparison**: A) COVID+ patients and healthy controls; B) Asymptomatic and symptomatic; C) Severe and moderate. The size of the circles is proportional to the strength of the correlation, and the colour represents the direction (colour legends are shown on the right), where large dark blue represents a strong negative correlation, and a large dark red circle represents a strong positive correlation. SDE miRNAs are on the horizontal axis and cytokines/chemokines on the vertical axis.

We also evaluated the change of cytokine and chemokine levels for all comparisons (**Supplementary Data 5**). Patients infected with SARS-CoV-2 showed higher levels of proinflammatory cytokines, which were increasing with COVID-19 severity. Later, we analyzed the correlation between SDE miRNAs and cytokines for each severity group contrast (Figure 2). We observed that several cytokines, chemokines, and growth factors significantly correlated with the dysregulated miRNAs resulting from each comparison (A, COVID-19 patients vs. healthy controls; B, asymptomatic vs. symptomatic; C, severe vs. moderate).

#### Characterization of COVID-19 infection

**Figure 3.**
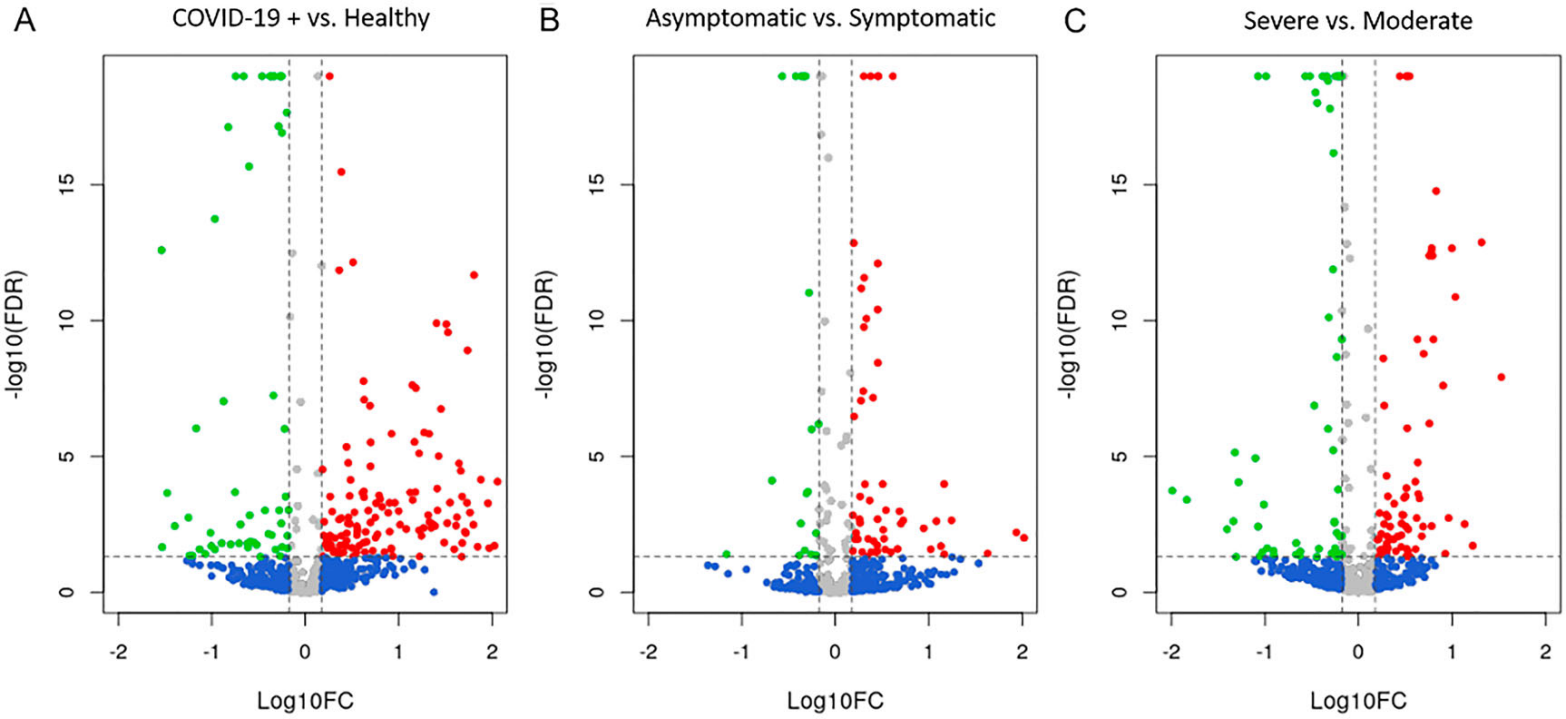
**Differential expression analysis of miRNAs.** Volcano plots showing the SDE miRNAs for each comparison: **A.** COVID+ vs healthy controls, **B**. AM vs symptomatic and **C**. Moderate vs severe. Red dots show miRNAs with a FDR corrected *p*-value ≤ 0.05 and a FC ≥ 1.5; Green dots show miRNAs with a FDR corrected *p*-value ≤ 0.05 and a FC ≤ −1.5; Blue dots represent miRNAs with a |FC| ≥ 1.5 that do not present statistical significance; Gray dots show miRNAs without statistical significance and a |FC| < 1.5. AM, asymptomatic; FDR, false discovery rate; FC, fold change.

We first studied the miRNAs expression differences between SARS-CoV-2 infected patients and healthy controls, which showed the presence of 200 SDE miRNAs (Figure 3A; **Supplementary Data 6**). In COVID-19 patients, 142 miRNAs were upregulated and 58 downregulated. SARS-CoV-2 infected patients had a strong upregulation of miRNAs such as the hsa-miR-4665-5p, hsa-miR-3190-3p, hsa-miR-331-3p, hsa-miR-4525, hsa-miR-431-5p, hsa-miR-6721-5p, hsa-miR-4661-5p, hsa-miR-548a-3p, hsa-miR-4745-5p, and hsa-miR-3150b-3p, which showed a FC > 100.

The functional analysis reported 29 Reactome biological pathways. The most relevant pathways were influenza infection, influenza viral RNA transcription and replication, viral mRNA translation, and estrogen-dependent gene expression, among others (**Supplementary Data 7; Supplementary Data 8**).

SDE miRNAs in COVID-19 patients were significantly correlated with proinflammatory cytokines such as IL-6, IL-12, IP10, and TNFα (Figure 2A**)**, while healthy and infected patients showed different correlation patterns (**Supplementary Data 9A and B, respectively**). The proinflammatory hsa-miR-320 family was highly expressed in SARS-CoV-2 infected patients, positively correlated with proinflammatory cytokines such as IL-12, IL-6, IL-8, IP10, MIG, TIM3, and TNFα, especially the hsa-miR-320b. These correlations were not observed in healthy controls, where most of the significant correlations with proinflammatory cytokines were negative (**Supplementary Data 9A**).

#### Asymptomatic COVID-19 patients

We found 75 SDE miRNAs between asymptomatic (asymptomatic and mild) and symptomatic (moderate and severe) COVID-19 patients (Figure 3B**; Supplementary Data 10**). Nineteen of them showed a higher expression in asymptomatic patients, and the hsa-miR-1291 was the most upregulated (FC = 14.6). In contrast, most of the SDE miRNAs were downregulated, showing nine of them over a 10-fold decrease (hsa-miR-8485, hsa-miR-1229-3p, hsa-miR-412-5p, hsa-miR-937-3p, hsa-miR-4750-5p, hsa-miR-296-5p, hsa-miR-26a-2-3p, hsa-miR-939-5p and hsa-miR-424-5p).

The functional pathway analysis showed that some of the most deregulated pathways were platelet aggregation (plug formation), IL-3, IL-5, and GM-CSF signalling, VEGFA-VEGFR2 pathway, and signalling by VEGF, among others (**Supplementary Data 11; Supplementary Data 12).** In line with these findings, asymptomatic patients showed lower levels of proangiogenic markers such as FGF2 and HGF than symptomatic patients, which were positively correlated with hsa-miR-4854 and hsa-miR-548t-5p, respectively (Figure 2). This latter miRNA showed a high positive correlation with IFNγ, IL-1b, IL-12, IL-1RA, and IL-4 exclusively in asymptomatic participants **(Supplementary Data 13A)**. Symptomatic patients showed a different correlation pattern, showing a high positive correlation of the hsa-miR-320 with ferritin, HGF, IL-12, IL-4, IL-6, IL-8, IP10, MIG, TIM3, and TNFα (**Figure 13B**).

#### Severe COVID-19 patients

We found 137 SDE miRNAs between severe and moderate COVID-19 patients (**Supplementary Data 14**). Seventy-seven miRNAs were upregulated in severe patients (Figure 3**C**), being the hsa-miR-3976, hsa-miR-4488, hsa-miR-3150b-3p, hsa-miR-7704 and hsa-miR-3168 the most upregulated (FC > 10). Moreover, 60 miRNAs showed a downregulation in severe patients (Figure 3**C**) and the most downregulated miRNAs with a 10-fold decrease (FC < 0.1) where the hsa-miR-7848-3p, hsa-miR-7110-3p, hsa-miR-1197, hsa-miR-4686, hsa-miR-374b-3p, hsa-miR-4473, hsa-miR-758-3p, hsa-miR-5004-3p, hsa-miR-146a-3p, hsa-miR-329-5p, hsa-miR-744-3p, hsa-miR-1285-5p and hsa-miR-6516-3p.

The pathway analysis indicated specific deregulation of several epigenetic pathways (PRC2 methylate histones and DNA, epigenetic regulation of gene expression, and SUMOylation of DNA methylation proteins) (**Supplementary Data 15; Supplementary Data 16**).

Severe patients showed an increase of proinflammatory cytokines, where the most elevated were IL-8, IL-6, IP10, MIG, MIP1, and TIM3, among others (**Supplementary Data 5**). The proinflammatory hsa-miR-320 family was highly expressed in severe patients (**Supplementary Data 14**), showing a high positive correlation with proinflammatory cytokines.

Conversely, the hsa-miR-26a-5p (a negative regulator of the inflammatory response) and the miR-98-5p (a member of the let-7 miRNA family) showed lower levels in severe patients and were negatively correlated with all proinflammatory cytokines (Figure 2**C**), (**Supplementary Data 17B**).

### Mortality predictive model

Characteristics of patients by mortality status are shown in **Supplementary Data 18**. We identified 12 miRNAs as predictors of 90-days mortality (hsa-let-7f-1-3p, hsa-let-7g-5p, hsa-miR-1255a, hsa-miR-140-3p, hsa-miR-20a-5p, hsa-miR-22-3p, hsa-miR-3180-3p, hsa-miR-3180, hsa-miR-363-5p, hsa-miR-4510, hsa-miR-548h-3p and hsa-miR-6130) (**Supplementary Data 19, 20A and 20B**), but two of them (hsa-miR-548h-3p and hsa-miR-3180) did not meet the filtering criteria (**Supplementary Data 19**). Among the ten selected miRNAs, only two were associated with a lower probability of survival at 90 days (hsa-miR-22-3p and hsa-miR-3180-3p), whereas eight were associated with a better prognosis (hsa-let-7f-1-3p, hsa-let-7g-5p, hsa-miR-1255a, hsa-miR-140-3p, hsa-miR-20a-5p, hsa-miR-363-5p, hsa-miR-4510, and hsa-miR-6130).

**Figure 4.**
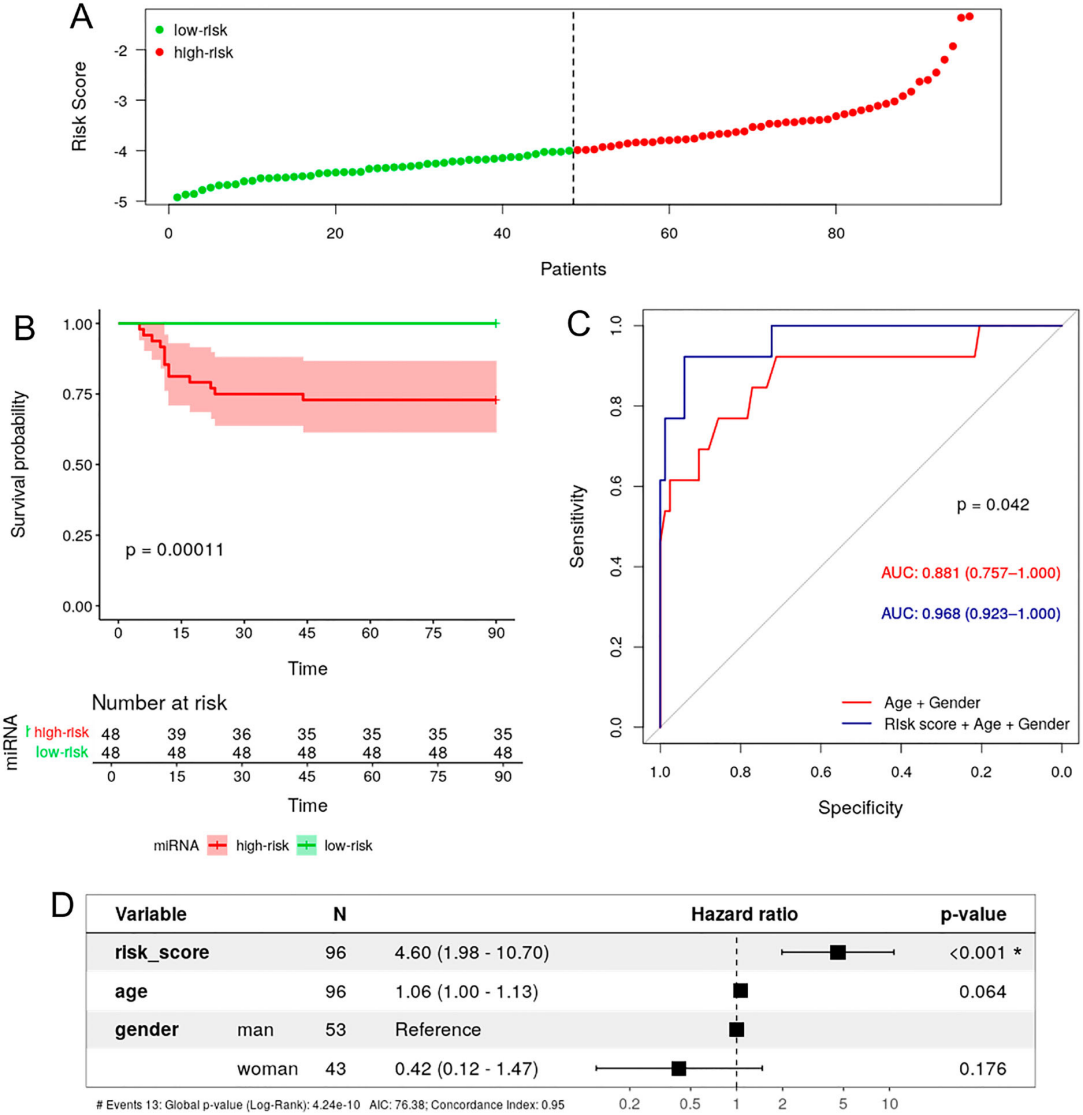
**Survival analysis of COVID-19 patients and 10-miRNA signature risk score**. **A.** Distribution of risk score values for low-risk and high-risk subjects. **B.** KM survival curve between the high-risk and low-risk groups stratified by median risk score. **C.** ROC curve of survival showing the prognostic ability (AUC) of age + gender (red) and miRNA risk score + age + gender (blue). **D.** Forest plot of the multivariate Cox regression analysis for age, gender and 10-miRNA risk score. The HR of each variable is indicated with 95% CI and statistical significance (*p*-value). KM, Kaplan-Meier; ROC, receiver operating characteristic; AUC, area under the curve; HR, hazard ratio; CI, confidence interval.

This ten miRNA-expression signature (**Supplementary Data 21**) was used to generate the miRNA-MRS, whose median value was −3.992, which was used as a threshold to stratify the study subjects into low-risk (risk score < −3.992, n = 42) and high-risk (risk score ≥ −3.992, n = 42) (Figure 4**A**). Kaplan-Meier analysis confirmed the significant differences in the overall mortality (OS) (*p*-value <0.001) (Figure 4**B**). Moreover, ROC curve analysis confirmed the predictive power of the miRNA-MRS, which was improved by adjusting with age and gender, showing a significantly higher AUROC value than the basic model considering only age and gender (0.968 vs. 0.881, *p*-value = 0.042), (Figure 4**C**). Additionally, we analyzed the diagnostic accuracy of adding the miRNA-MRS to the model with age and gender by calculating sensitivity, specificity, and positive and negative predictive value. Considering the cut-off for maximum sensitivity and specificity, the model showed sensitivity and specificity values above 90% (**Supplemental Data 22**). Additionally, results were confirmed by the multivariate Cox-regression model, highlighting the independent prognostic value of the risk-score (hazard ratio (HR) = 4.599; 95% confidence interval (CI) (1.977− 10.700), *p*-value <0.001) (Figure 4**D**).

## Discussion

Our results showed that SARS-CoV-2 infection produces a strong disturbance of miRNA profile from the onset of the disease, exhibiting at this time a specific miRNA signature for each COVID-19 severity. The identified miRNAs could be used as early severity markers at hospital admission. To our knowledge, this is the first large-scale study that characterizes the expression profile of plasma miRNAs in COVID-19 patients of different clinical statuses, identifying specific miRNA signature for each severity group and a specific miRNA mortality risk score (miRNA-MRS).

### miRNA altered profile by SARS-CoV-2 infection

We identified 200 SDE miRNAs between COVID-19 patients and healthy controls, most of them upregulated in SARS-CoV-2 infected patients. Although scarce studies on human miRNAs during COVID-19 have been published, some of the most upregulated miRNAs in COVID-19 patients have been identified to target the SARS-CoV-2 genome, which supports their functionality. This is the case of hsa-miR-4665-5p (predicted binding site in SARS-CoV-2 genome) [19], hsa-miR-3190-3p (predicted targeting of ORF1ab and ORF8 genes) [20], hsa-miR-4745-5p (expressed in lung tissue with affinity to the 3′UTR of SARS-COV-2 genome) [21] and hsa-miR-3150b-3p (predicted binding site in the leader sequence of SARS-CoV-2) [22]. Remarkably, other strongly dysregulated miRNAs, such as hsa-miR-431-5p, hsa-miR-548a-3p, and hsa-miR-331-3p have been previously linked to several viral infections, including ZIKV and HBV [23,24]. It has also been suggested that hsa-miR-431-5p plays a role in restricting HCV replication after its induction by IFNβ [25]. Due to the known antiviral activity of IFNβ, constituting the first line of defence against viral infections, IFNβ-regulated miRNAs could be used as early biomarkers of COVID-19 disease, being detected before the traditional markers of immune activation or inflammation.

Disrupted miRNAs in COVID-19 patients were strongly involved in biological processes related to viral infections, including influenza viral RNA transcription and replication or viral mRNA translation, suggesting a direct binding/effect of the SDE miRNAs on the viral genome. Remarkably, biological pathways involving eukaryotic mRNA translation also appeared as dysregulated, including cap-dependent translation initiation, translation initiation complex formation, elongation, and termination, among others. These functional alterations agree with *in vitro* studies that point to the inhibition of cellular mRNA expression in cells infected with different coronaviruses, including SARS-CoV-2 [26]. Coronaviruses seem to hijack the host translational machinery, thus favouring viral protein production. In fact, Kim *et al.* showed that host gene expression is almost suppressed in the first hours of infection [27], which remarks the early disturbance of miRNA machinery, as miRNAs are the master regulators of gene expression.

Additionally, certain trace elements pathways such as metabolism and transport of copper (Cu) and selenium (Se) appeared also disrupted. These elements are essential for a normal immune response, and their serum levels have been proposed as good predictors of COVID-19 survival [28].

However, the most dysregulated was the estrogen signalling pathway. Estrogen plays a crucial role in innate and adaptive immune responses, showing an immunoenhancing effect. Estrogen receptors are involved in tissue repair processes during respiratory virus infection [29]. A weaker estrogen receptor signalling is linked to a poor evolution of influenza, while estrogen treatment help to control the inflammatory reactions and improve survival [30]. An anti-inflammatory effect of estrogens in COVID-19 has been suggested, reducing SARS-CoV-2 infectivity through the regulation of proinflammatory signalling pathways [31]. This evidence, along with our results and ongoing clinical trials [32], point to the necessity of continuing to investigate the effects of these molecules in COVID-19, to discover the potential use of estrogens as therapy or preventive strategy against COVID-19.

### miRNA profile of asymptomatic infection

We identified a signature of 75 SDE miRNAs in asymptomatic with respect to symptomatic patients that could be potentially used as early predictors of a favourable clinical course. Some of the most upregulated miRNAs in symptomatic patients have previously been related to viral infections, including influenza A viruses (IAVs), HIV1, and HIV2 viruses [33,34]. The hsa-miR-424-5p targets different RNA viruses, including SARS-CoV-2, and the hsa-miR-939-5p targets the SARS-CoV-2 genome 3′UTR [21]. Additionally, the hsa-miR-939-5p regulates several anti-inflammatory genes, including *IL-6, VEGFA, TNFα, NFκB2, and NOS2A*, showing a key role as a mediator in inflammatory networks [35].

We also observed up to 19 upregulated miRNAs in asymptomatic patients. Among the most upregulated miRNAs, hsa-miR-708-3p and hsa-miR-1291 were recently found by Chen *et al.* as differentially expressed between patients with mild and severe symptoms of COVID-19 [36]. The hsa-miR-1291 was more than 14 times expressed in our asymptomatic patients. This miRNA acts upstream of the chemokine receptor CCR2, which is known to induce monocyte and macrophage recruitment to sites of inflammation and regulates the immune response by controlling the proportion of effector and regulatory T cells [36]. An increased *CCR2* expression has been reported in patients with severe COVID-19 [37], and similarly, hsa-miR-1291 has been pinpointed as a potential early biomarker of COVID-19 severity [36]. Therefore, tight control of inflammation by hsa-miR-1291 could be behind the COVID-19 control in asymptomatic patients.

Remarkably, asymptomatic patients showed a higher expression of hsa-miR-150-5p, a master regulator of the immune system with crucial roles in the development of immune response, including respiratory viral infections [38]. Hsa-miR-150-5p is involved in the modulation of B cell differentiation, development of natural killer (NK), and invariant NK T (iNKT) cells by targeting the transcription factor c-Myb [39], among others. Critically ill patients showed decreased levels of hsa-miR-150-5p, and its expression is inversely correlated with the number of days of ICU stay [37]. Thus, a higher expression of hsa-miR-150-5p could lead to a fine-tune immune response to successfully clear the SARS-CoV-2 virus without an exaggerated immune response.

The functional analysis further confirmed these results, which showed inflammatory and immune processes as the most dysregulated biological events by the SDE miRNAs. In fact, we observed disruption of platelet aggregation, interleukin-3, −5 and GM-CSF signalling, VEGFA-VEGFR2, and signalling by VEGF. Although we could not evaluate VEGF, higher plasma levels of other angiogenic growth factors such as HGF and FGF were observed in asymptomatic patients. Moreover, a strong enrichment of phagocytic processes, including FcγRIIIa-mediated phagocytosis, regulation of actin dynamics for phagocytic cup formation, and Fcgamma receptor (FCγR) dependent phagocytosis was observed. Phagocytosis is one of the main processes in innate immune response, contributing to the fight against infectious agents, as it has been detected in COVID-19 severe patients [40]. The FCγR is also involved in cell activation and increased proinflammatory cytokine production [41], which could be promoting the outbreak of COVID-19 cytokine storm.

### Early miRNA signature predicting the severity of COVID-19

Certain risk factors linked to a more severe course of COVID-19 have already been identified, such as age or male gender. However, these factors cannot predict the evolution of the disease by themselves, and it remains essential to find additional biological factors decisive to trigger COVID-19 severity. In this sense, we identified the molecular players in the early stages that could be behind the progression to a severe COVID-19.

We identified 137 SDE miRNAs between severe patients and those with moderate symptoms. Seventy-seven of these miRNAs were upregulated in severe patients, with hsa-miR-3976, hsa-miR-4488, hsa-miR-3150b-3p, hsa-miR-7704, and hsa-miR-3168 showing the strongest disruption. Most of them showed some evidence of playing a role in different viral infections [42,43]. Moreover, hsa-miR-3150b-3p and hsa-miR-7704 are putative targets to the leader sequence and *ORF3a* gene of the SARS-CoV-2 genome, respectively [20,22]. Meanwhile, 60 miRNAs showed downregulation in severe patients. Again, some of the strongly disrupted miRNAs potentially target the SARS-CoV-2 genome, such as hsa-miR-1197, hsa-mir-5004-3p, and hsa-miR-1285-5p [19,44], which could be involved in inhibiting virus replication. In this regard, our functional enrichment results suggest a strong involvement of SDE miRNAs in biological processes such as viral RNA transcription, replication and translation, further supporting this hypothesis. Several examples of host miRNA implication on RNA viruses replication and pathogenesis have been described, as these viruses may modify miRNAs profile within the cell to establish a proviral environment [45]. Our data suggest that the altered miRNA profile observed in COVID-19 patients could lead to a differential viral load according to the severity of the disease. Similarly, RNAemia and viral load on admission have been related to mortality [46]. However, future studies would be necessary to clarify the interplay between miRNAs, RNAemia and viral load.

Additionally, we observed a strong disturbance of miRNAs that modulate the immune response, suggesting that the differential severity status could be a result of both the altered regulation of viral translation/replication processes but also the differential modulation of immune / inflammatory responses following infection.

In fact, other strongly downregulated miRNAs in severe patients directly regulate antiviral response, such as the hsa-miR-744-3p, against IAV and RSV through targeting MAPK-activated protein kinase 2 [47]. On the contrary, hsa-miR-758-3p downregulates the expression of the viral RNA receptors Toll-like receptors 3 (TLR3) and 7 (TLR7) following HCV infection [48]. Additionally, we observed a deep downregulation of hsa-miR-146a-3p, involved in several autoimmune and inflammatory diseases such as cystic fibrosis, where it controls the IL-6 production, contributing to the restriction of the inflammatory response [49]. Thus, the reduced expression of this miRNA in severe patients is related to an increase in IL-6 production, which has been reported in severe and critical COVID-19 patients, where IL-6 acts as a vital amplifier [50]. Besides, we also observed an increase in additional proinflammatory cytokines such as IL-8, IP10, MIG, MIP-1, and TIM3, among others.

The dysregulated miRNAs in severe patients were mainly involved in epigenetic pathways, which have been reported as altered in COVID-19 [51].

### Ten miRNA-based risk score as a predictor of COVID-19 mortality

The overall mortality rate of COVID-19 is around 1-3% in most countries, and it is hugely affected by certain epidemiological factors, such as age, gender, and comorbidities [52]. However, these estimates are highly dependent on the countries healthcare systems and vaccination progress. While no differences in comorbidities or treatments by mortality status were observed in this study, we identified early biomarkers that allowed predicting an increased risk of death. We screened 767 miRNAs using a LASSO Cox regression model and identified a signature of ten SDE miRNAs that can be used as good predictors of COVID-19 mortality.

While little is known about the potential role of hsa-miR-1255a and hsa-miR-6130 in COVID-19 disease, some of the selected miRNAs have already been predicted to target the SARS-CoV-2 genome, including hsa-let-7g-5p, hsa-miR-363-5p, and hsa-miR-4510 [20]. Moreover, a decreased hsa-miR-20a-5p expression in peripheral blood was observed in patients with COVID-19 [53]. This miRNA has been computationally predicted to target several proteins that may play a role in SARS-CoV-2 induced cell death, including SMAD3, SMAD4, and TGFBR1 [54]. In fact, the binding of SARS-CoV N protein to SMAD3 has been shown to interfere with the complex formation between SMAD3 and SMAD4, thus inhibiting the apoptosis of SARS-CoV-infected cells mediated by the TGF-signalling pathway [55], in favour of virus packaging and replication.

A link between COVID-19 disease and the miRNAs hsa-let-7f-1-3p and hsa-miR-140-3p has also been reported, showing a downregulation in cells infected with SARS-CoV-2 [19,56]. Kim *et al.* proposed that hsa-miR-140-3p targets the serine protease TMPRSS2, which processes the viral S protein that binds to the ACE2 host receptor [56]. Moreover, several studies have described its potential to modulate Bcl-2 expression, which has an essential role in regulating several cellular processes that could have an impact on viral infection progression [57].

Although both age and gender have been widely reported as risk factors for the greater severity and mortality of COVID-19, we found that the sensitivity and specificity in the prediction of COVID-19 mortality significantly increased when including the ten-miRNA signature-based risk score in our model. Moreover, we confirmed the independent predictive value of the risk-score by a multivariate Cox-regression model including both epidemiological variables.

Although further research is still needed to decipher the biological mechanisms that could be behind the potential effect of these human miRNAs in COVID-19 mortality, our results provide a new promising diagnostic tool that could contribute to the improved management of patients with a higher risk of death at disease onset.

This miRNA-MRS could be implemented in routine diagnostics through an amplification-based detection method such as multiplex real time quantitative PCR, or even through emerging technologies such as hybridization-based detection methods [58]. In this latter case, low amount of starting material and no reverse transcription or cDNA amplification is needed, which make this medium-throughput technology highly suitable for a scalable use [59]. These systems can be easily combined with an analysis software to give a simple readout indicating the disease probability score based on the concentration of each miRNA [60].

Nevertheless, this study has several limitations. First, our cohort included a limited number of deaths among participants. Thus, we decided to include all the study samples as a training set in order to obtain more reliable and representative results. Therefore, our findings would need to be validated in an external cohort of COVID-19 patients. Nevertheless, at the time of manuscript submission, there were no high-throughput miRNA sequencing data available from studies of COVID-19 patients reporting mortality analysis results to perform the validation of our miRNA-based model. Moreover, most of the participants in our cohort were Caucasian and Hispanic, while other ethnic groups were underrepresented. Due to the differences in the genetic background and miRNA expression, the predictive value of the miRNA-based risk score should also be confirmed in studies with ethnically more diverse populations. Additionally, it would also be interesting to study whether miRNA profiles correlate with SARS-CoV-2 viral load or RNAemia. However, we were unable to analyze it because no remaining sample was available. Finally, further studies would be needed to explore how the duration of illness influences the microRNA profiles.

## Conclusion

SARS-CoV-2 infection deeply disturbs the plasma miRNA expression profile from an early stage of COVID-19, making miRNAs highly valuable as early predictors of severity and mortality.

## Supplementary Material

Supplemental Material
