# Supplementary material for: Plasma miRNA profile at COVID-19 onset predicts severity status and mortality
Source: Emerg Microbes Infect. 2022 Feb 27;11(1):676–88. doi: 10.1080/22221751.2022.2038021 (PMC8890551; doi:10.1080/22221751.2022.2038021)
Supplement: Supplemental Material [file TEMI_A_2038021_SM5147.docx]

**INDEX**

# **SUPPLEMENTARY DATA**

[**Supplementary Data 1**](#_Supplementary_Data_1:): Study flow diagram.

[**Supplementary Data 2**](#_Supplementary_Data_2.)**:** Data Processing Pipeline: Bioinformatics Analysis.

[**Supplementary Data 3**](#_Supplementary_Data_3:): Custom Procartaplex multiplex immunoassay (Invitrogen) panel.

[**Supplementary Data 4**](#_Supplementary_Data_4.)**:** Extended statistical analysis information.

[**Supplementary Data 5**](#_Supplementary_Data_5:)**:** Differences in plasma cytokine and chemokine levels according to severity status: i) COVID+ vs. healthy; ii) asymptomatic vs. symptomatic; iii) severe vs. moderate.

[**Supplementary Data 6**](#_Supplementary_Data_6:)**:** Significant differentially expressed (SDE) miRNAs between COVID+ patients and healthy controls.

[**Supplementary Data 7**](#_Supplementary_Data_7:)**:** miRNA network of COVID+: miRNA– targeted genes interaction with significantly enriched pathways for SDE miRNAs between COVID+ patients and healthy controls.

[**Supplementary Data 8**](#_Supplementary_Data_8:)**:** miRNA-targeted pathways for significant differentially expressed (SDE) miRNAs of COVID+ patient’s vs. healthy controls.

[**Supplementary Data 9**](#_Supplementary_Data_9:)**:** Spearman correlation plot showing the correlation between SDE miRNAs and plasma cytokines / chemokines for: A) healthy controls and B) COVID+ patients.

[**Supplementary Data 10**](#_Supplementary_Data_10:)**:** Significant differentially expressed (SDE) miRNAs between asymptomatic and symptomatic COVID+ patients.

[**Supplementary Data 11**](#_Supplementary_Data_11:)**:** miRNA network of COVID+ asymptomatic patients: miRNA– targeted genes interaction with significantly enriched pathways for SDE miRNAs between asymptomatic and symtomatic patients.

[**Supplementary Data 12**](#_Supplementary_Data_12:)**:** miRNA-targeted pathways for SDE miRNAs of asymptomatic patients vs. symptomatic.

[**Supplementary Data 13**](#_Supplementary_Data_13:)**:** Pearson correlation plot showing the correlation between SDE miRNAs and plasma cytokines / chemokines for: A) Asymtomatic and B) Symptomatic.

[**Supplementary Data 14**](#_Supplementary_Data_14:)**:** Significant differentially expressed (SDE) miRNAs between severe and moderate COVID+ patients.

[**Supplementary Data 15**](#_Supplementary_Data_15.)**:** miRNA network of COVID+ severity: miRNA– targeted genes interaction with significantly enriched pathways for SDE miRNAs between severe and moderate patients.

[**Supplementary Data 16**](#_Supplementary_Data_16:)**:** miRNA-targeted pathways for significant differentially expressed (SDE) miRNAs of severe vs. moderate patients.

[**Supplementary Data 17**](#_Supplementary_Data_17:)**:** Pearson correlation plot showing the correlation between SDE miRNAs and plasma cytokines / chemokines for: A) Moderate and B) Severe patients.

[Supplementary Data 18](#_Supplementary_Data_18:): Clinical, epidemiological and virological characteristics of SARS-CoV-2 infected patients by mortality

[**Supplementary Data 1**](#_Supplementary_Data_18:)**9:** miRNA mortality Risk Score (miRNA-MRS) results.

**Supplementary Data 20:** Mortality analysis with LASSO Cox regression model.

[**Supplementary Data 2**](#_Supplementary_Data_20:)**1:** Forest plot displaying the 10 miRNAs included in the final miRNA Risk Score model.

[**Supplementary Data 2**](#_Supplementay_Data_21.)**2:** Diagnostic accuracy of the model including the miRNA-MRS in combination with age and gender for predicting COVID-19-related death at 90 days.

**SUPPLEMENTARY DATA**

## **Supplementary Data 1**: Study flow diagram.


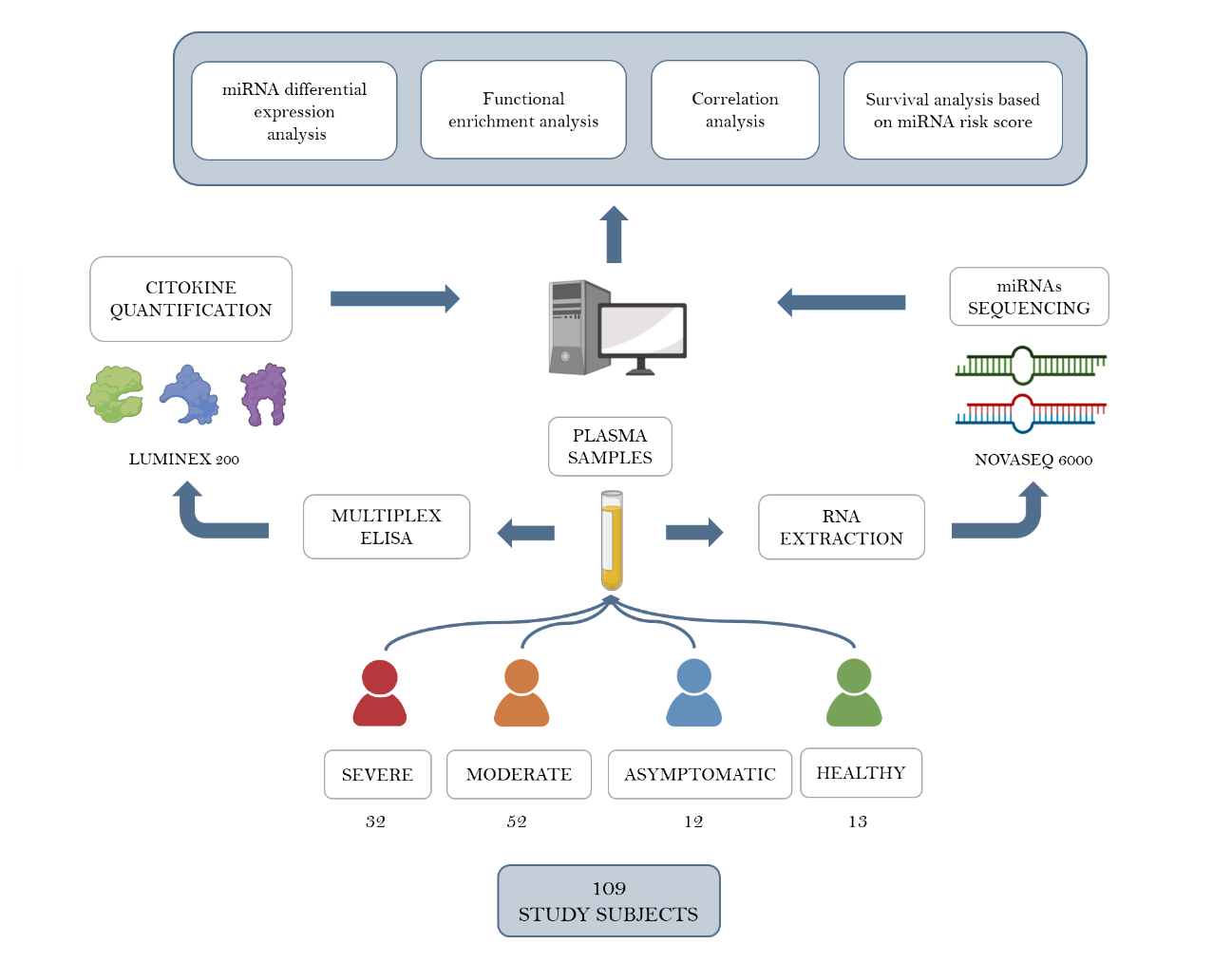


## **Supplementary Data 2**. Data Processing Pipeline: Bioinformatics Analysis

Sequence data from BCL files were converted to FASTA with bcl2fastq (Illumina). Samples were demultiplexed and each read was assigned to the correct sample. Raw data was then analyzed with a specific bioinformatic pipeline for the identification and quantification of known miRNAs.

First, an initial filtering step was performed to remove reads with ambiguous base calls, which did not meet the Ilumina chastity filter based on quality measures. Remaining reads were quality checked using FastQC (v0.11.3) (1) and adapter sequences were trimmed with cutadapt (v1.13) (2). Next, reads were processed with miRDeep2 (v.0.0.7). This software allows the alignment of the reads to the reference human genome (GRCh38) using the mapper.pl module based on Bowtie1. Only the alignments with zero mismatches in the seed region and those that did not map to more than five different loci in the genome were retained.

Quantification of the miRNAs was performed with the quantifier.pl module, which determines the expression of the corresponding known miRNAs in two steps. First, predefined mature miRNA sequences are mapped against the predefined precursors in miRBase (v2.0). the public repository for all published miRNA sequences (3). Second, sequencing reads are mapped against the precursor sequences. Reads falling into an interval of two nucleotides upstream and five nucleotides downstream of the mature miRNA sequences were determined for quantification.

## **Supplementary Data 3: Custom Procartaplex multiplex immunoassay (Invitrogen) panel.**

| **Target Name** | **Full description** |
| --- | --- |
| D-dimer | D-dimer |
| Ferritin | Ferritin |
| FGF-2 | Fibroblast growth factor 2 |
| G-CSF (CSF-3) | Colony stimulating factor 3 |
| HGF | Hepatocyte growth factor |
| IFN gamma | Interferon gamma |
| IL-1 beta | Interleukin-1 beta |
| IL-10 | Interleukin-10 |
| IL-12 / IL-23p40 | Interleukin-12 |
| IL-13 | Interleukin-13 |
| IL-15 | Interleukin-15 |
| IL-1RA | Interleukin-1 receptor antagonist |
| IL-2 | Interleukin-2 |
| IL-4 | Interleukin-4 |
| IL-6 | Interleukin-6 |
| IL-7 | Interleukin-7 |
| IL-8 (CXCL8) | Interleukin-8 |
| IP-10 (CXCL10) | C-X-C motif chemokine ligand 10 |
| M-CSF (CSF1) | Colony stimulating factor 1 |
| MCP-1 (CCL2) | C-C motif chemokine ligand 2 |
| MCP-3 (CCL7) | C-C motif chemokine ligand 7 |
| MIG (CXCL9) | C-X-C motif chemokine ligand 9 |
| MIP-1 alpha (CCL3) | C-C motif chemokine ligand 3 |
| NTproBNP |  |
| TIM-3 (HAVCR2) | Hepatitis A virus cellular receptor 2 |
| TNF alpha | Tumor necrosis factor alpha |

## **Supplementary Data 4. Extended statistical analysis information**

For descriptive data, differences between groups were assessed by Kruskal-Wallis test for continuous variables and Pearson’s chi-squared test for discrete variables.

MiRNA counts were normalized with DESeq2 method (v1.30.0), after filtering those with low read counts using *FilterByExpr*(). The miRNA expression differences between severity groups were analyzed using a binomial negative generalized linear model (bnGLM) adjusting by gender and age. MiRNAs with fold change (FC) ≥ 1.5 and an adjusted p-value ≤ 0.05 using the false discovery rates (FDR) were considered significant differentially expressed (SDE) miRNAs.

For the mortality analysis, mortality time was defined as time between hospitalization and death, and individuals alive at 90 days were considered censored data. Variance stabilizing transformation (VST) normalized counts of all identified miRNAs were included in a least absolute shrinkage and selection operator (LASSO) multivariate Cox proportional hazards regression model to select the miRNAs with best prognostic value (R package glmnet 4.1-3). The optimal lambda parameter (largest value of lambda with an error within 1 standard error (SE) of the minimum (lambda.1SE) was selected based on 10-fold cross-validation (logarithmic scale). Each of the selected miRNAs was tested for the proportional hazards assumption using the cox.zph() function. A miRNA mortality risk score (miRNA-MRS) was estimated as a linear combination of the expression of selected miRNAs weighted by Cox regression coefficients. Patients were then classified into high and low risk-score mortality groups, with the median risk-score value as a cutoff. Kaplan-Meier curves were generated to evaluate the survival profiles of both patient groups and the log-rank test assayed differences between groups (R package survminer 0.4.9). Next, we analysed the diagnostic performance of miRNA-MRS for predicting COVID-19-related death using the area under the receiver-operating characteristic (AUROC) curve. Delong test was carried out to compare the AUROC curves. Additionally, we analyzed the diagnostic accuracy of adding the miRNA-MRS to the model with age and gender by calculating sensitivity, specificity and positive and negative predictive value, taking into account the cut-off for maximum test sensitivity plus specificity. A multivariate Cox-regression was performed to further confirm the independent prognostic value of the risk-score (R package survival 3.2-10).

Cytokine and chemokine concentration were transformed by weighted Box-Cox followed by plate-normalization by quantiles as previously described (14). Comparisons between severity groups was performed with a GLM with gamma distribution. Correlation analysis using a Spearman correlation test (q-value <0.1) was carried out to investigate the relationship between miRNA counts and cytokine/chemokine levels.

Two-sided tests were used for all statistical methods. Statistical software R (v4.0.3) was used for all statistical analyses.

## **Supplementary Data 5:** Differences in plasma cytokine and chemokine levels according to severity status. Statistically significant values are highlighted in bold.

1. COVID+ vs. healthy

| **Plasma markers** | **Log2 ratio** | **p-value** | **FDR** |
| --- | --- | --- | --- |
| IL-15 | 0.13 | <0.001 | **<0.001** |
| IL-1RA | 0.05 | <0.001 | **0.003** |
| NTproBNP | 0.18 | <0.001 | **0.003** |
| MCP-3 | 0.07 | 0.001 | **0.006** |
| IP-10 | 0.23 | 0.003 | **0.015** |
| HGF | 0.11 | 0.003 | **0.015** |
| FGF-2 | 0.05 | 0.027 | **0.094** |
| IL-2 | 0.08 | 0.029 | 0.094 |
| G-CSF | 0.05 | 0.054 | 0.148 |
| IL-1 beta | 0.07 | 0.057 | 0.148 |
| IL-10 | 0.05 | 0.081 | 0.191 |
| IL-7 | 0.06 | 0.096 | 0.192 |
| TNF alpha | 0.05 | 0.094 | 0.192 |
| MIP-1 alpha | 0.06 | 0.147 | 0.273 |
| IL-6 | 0.07 | 0.165 | 0.285 |
| IL-8 | 0.04 | 0.232 | 0.377 |
| Ferritin | 0.10 | 0.256 | 0.392 |
| MIG | -0.06 | 0.285 | 0.412 |
| IL-12 | 0.02 | 0.353 | 0.483 |
| D-dimer | 0.07 | 0.451 | 0.586 |
| M-CSF | -0.02 | 0.500 | 0.619 |
| IFN gamma | -0.01 | 0.726 | 0.858 |
| TIM-3 | -0.01 | 0.830 | 0.939 |
| IL-13 | 0.00 | 0.955 | 0.982 |
| IL-4 | 0.00 | 0.959 | 0.982 |
| MCP-1 | 0.00 | 0.982 | 0.982 |

1. Asymptomatic vs. symptomatic

| **Plasma marker** | **Log2ratio** | | **p-value** | **FDR** |
| --- | --- | --- | --- | --- |
| FGF-2 | | -0.06 | 0.002 | **0.053** |
| HGF | | -0.11 | 0.005 | **0.061** |
| TNF alpha | | -0.07 | 0.018 | 0.157 |
| IL.1RA | | -0.03 | 0.073 | 0.379 |
| IL-13 | | -0.08 | 0.064 | 0.379 |
| IL-15 | | -0.04 | 0.092 | 0.397 |
| IL-10 | | -0.04 | 0.205 | 0.481 |
| IL-1 beta | | -0.05 | 0.218 | 0.481 |
| IL-2 | | -0.04 | 0.264 | 0.481 |
| IL-4 | | -0.04 | 0.278 | 0.481 |
| IL-6 | | -0.07 | 0.133 | 0.481 |
| IP-10 | | -0.09 | 0.249 | 0.481 |
| M-CSF | | 0.04 | 0.225 | 0.481 |
| MIG | | 0.06 | 0.239 | 0.481 |
| MIP-1 alpha | | -0.06 | 0.151 | 0.481 |
| G-CSF | | -0.03 | 0.349 | 0.506 |
| MCP-3 | | -0.02 | 0.350 | 0.506 |
| NTproBNP | | -0.05 | 0.343 | 0.506 |
| IL-7 | | -0.03 | 0.456 | 0.608 |
| TIM-3 | | 0.02 | 0.468 | 0.608 |
| Ferritin | | -0.04 | 0.679 | 0.802 |
| IFN gamma | | -0.02 | 0.667 | 0.802 |
| MCP-1 | | -0.01 | 0.761 | 0.861 |
| IL-8 | | 0.00 | 0.888 | 0.962 |
| IL-12 | | 0.00 | 0.947 | 0.985 |
| D-dimer | | 0.00 | 0.991 | 0.991 |

1. Severe vs. moderate.

| **Plasma marker** | **Log2ratio** | **p-value** | **FDR** |
| --- | --- | --- | --- |
| HGF | 0.10 | <0.001 | **0.002** |
| IL-8 | 0.10 | <0.001 | **0.002** |
| IL-6 | 0.13 | <0.001 | **0.002** |
| IP-10 | 0.21 | <0.001 | **0.002** |
| Ferritin | 0.22 | 0.001 | **0.003** |
| MIG | 0.13 | 0.001 | **0.003** |
| MIP-1 | 0.09 | 0.001 | **0.005** |
| TIM-3 | 0.06 | 0.006 | **0.020** |
| D-Dimer | 0.19 | 0.009 | **0.023** |
| TNF alpha | 0.05 | 0.008 | **0.023** |
| MCP-1 | 0.09 | 0.012 | **0.028** |
| NTproBNP | 0.10 | 0.013 | **0.028** |
| IL-10 | 0.05 | 0.014 | **0.028** |
| IL-4 | 0.07 | 0.020 | **0.037** |
| FGF-2 | 0.03 | 0.035 | 0.060 |
| M-CSF | 0.05 | 0.039 | 0.064 |
| IFN gamma | 0.05 | 0.078 | 0.118 |
| IL-12 | 0.02 | 0.082 | 0.118 |
| G-CSF | 0.03 | 0.157 | 0.210 |
| IL-2 | 0.04 | 0.170 | 0.210 |
| IL-7 | 0.04 | 0.169 | 0.210 |
| IL-1RA | 0.01 | 0.335 | 0.396 |
| IL-1b | 0.03 | 0.353 | 0.399 |
| MCP-3 | 0.01 | 0.414 | 0.448 |
| IL-15 | 0.00 | 0.841 | 0.874 |
| IL-13 | 0.00 | 0.904 | 0.904 |

## **Supplementary Data 6:** Significant differentially expressed (SDE) miRNAs between COVID+ patients and healthy controls (absolute fold change (FC) ≥ 1.5 and FDR ≤ 0.05).

| **MiRNA** | **FC** | **p-value** | **FDR** |
| --- | --- | --- | --- |
| hsa-miR-4665-5p | 388.04 | <0.001 | <0.001 |
| hsa-miR-3190-3p | 318.00 | <0.001 | <0.001 |
| hsa-miR-331-3p | 290.47 | <0.001 | <0.001 |
| hsa-miR-4525 | 243.99 | <0.001 | <0.001 |
| hsa-miR-431-5p | 187.49 | <0.001 | <0.001 |
| hsa-miR-6721-5p | 183.59 | <0.001 | <0.001 |
| hsa-miR-4661-5p | 175.41 | <0.001 | <0.001 |
| hsa-miR-548a-3p | 163.74 | <0.001 | <0.001 |
| hsa-miR-4745-5p | 114.05 | <0.001 | <0.001 |
| hsa-miR-3150b-3p | 105.63 | 0.005 | 0.020 |
| hsa-miR-5588-5p | 93.26 | 0.006 | 0.024 |
| hsa-miR-873-5p | 90.15 | <0.001 | 0.001 |
| hsa-miR-4511 | 75.52 | <0.001 | <0.001 |
| hsa-miR-216b-5p | 69.90 | 0.005 | 0.021 |
| hsa-miR-548ae-5p | 63.80 | <0.001 | <0.001 |
| hsa-miR-6786-3p | 62.98 | 0.001 | 0.003 |
| hsa-miR-627-5p | 57.90 | <0.001 | 0.001 |
| hsa-miR-5010-5p | 54.46 | <0.001 | <0.001 |
| hsa-miR-7849-3p | 53.76 | <0.001 | 0.001 |
| hsa-miR-6868-3p | 52.20 | 0.001 | 0.007 |
| hsa-miR-6804-5p | 51.07 | 0.001 | 0.006 |
| hsa-miR-4738-3p | 47.77 | 0.004 | 0.015 |
| hsa-miR-1271-5p | 47.68 | <0.001 | <0.001 |
| hsa-miR-6089 | 47.17 | 0.015 | 0.050 |
| hsa-miR-4489 | 46.14 | <0.001 | <0.001 |
| hsa-miR-937-3p | 44.14 | <0.001 | <0.001 |
| hsa-miR-6735-5p | 41.87 | <0.001 | 0.002 |
| hsa-miR-7110-5p | 40.94 | 0.001 | 0.004 |
| hsa-miR-3140-3p | 39.24 | 0.007 | 0.026 |
| hsa-miR-379-3p | 35.35 | <0.001 | 0.001 |
| hsa-miR-548ad-5p | 33.56 | <0.001 | <0.001 |
| hsa-miR-7977 | 33.11 | <0.001 | 0.003 |
| hsa-miR-6850-5p | 32.29 | <0.001 | <0.001 |
| hsa-miR-4467 | 30.68 | 0.003 | 0.015 |
| hsa-miR-3173-5p | 28.29 | <0.001 | <0.001 |
| hsa-miR-4659a-3p | 26.67 | <0.001 | <0.001 |
| hsa-miR-548q | 25.99 | <0.001 | 0.001 |
| hsa-miR-429 | 25.76 | <0.001 | <0.001 |
| hsa-miR-548ay-5p | 25.39 | <0.001 | <0.001 |
| hsa-miR-6511a-3p | 24.41 | 0.001 | 0.004 |
| hsa-miR-3180-3p | 22.90 | 0.001 | 0.003 |
| hsa-miR-3180 | 22.90 | 0.001 | 0.003 |
| hsa-miR-4662a-5p | 22.22 | 0.001 | 0.004 |
| hsa-miR-6873-3p | 21.28 | <0.001 | 0.003 |
| hsa-miR-195-3p | 21.09 | <0.001 | <0.001 |
| hsa-miR-98-3p | 20.63 | <0.001 | 0.001 |
| hsa-miR-933 | 18.72 | <0.001 | <0.001 |
| hsa-miR-8060 | 18.46 | 0.001 | 0.004 |
| hsa-miR-196a-5p | 17.49 | 0.002 | 0.008 |
| hsa-miR-671-5p | 16.73 | 0.015 | 0.048 |
| hsa-let-7f-2-3p | 16.53 | <0.001 | <0.001 |
| hsa-miR-581 | 16.49 | 0.001 | 0.005 |
| hsa-miR-380-5p | 15.24 | <0.001 | <0.001 |
| hsa-miR-6516-3p | 15.06 | <0.001 | <0.001 |
| hsa-miR-548e-3p | 14.72 | <0.001 | <0.001 |
| hsa-miR-1273h-5p | 14.08 | <0.001 | <0.001 |
| hsa-miR-10399-3p | 13.94 | <0.001 | <0.001 |
| hsa-miR-197-5p | 13.26 | <0.001 | <0.001 |
| hsa-miR-885-3p | 12.02 | 0.001 | 0.005 |
| hsa-miR-1287-5p | 10.34 | 0.001 | 0.003 |
| hsa-miR-4659b-3p | 9.98 | <0.001 | 0.001 |
| hsa-miR-7854-3p | 9.07 | <0.001 | 0.001 |
| hsa-miR-4658 | 8.64 | 0.014 | 0.047 |
| hsa-miR-361-5p | 8.37 | <0.001 | <0.001 |
| hsa-miR-4685-3p | 8.32 | 0.007 | 0.026 |
| hsa-miR-6862-5p | 8.29 | 0.007 | 0.024 |
| hsa-miR-3143 | 7.99 | 0.001 | 0.006 |
| hsa-miR-3605-5p | 7.96 | <0.001 | 0.001 |
| hsa-miR-10527-5p | 7.71 | <0.001 | 0.001 |
| hsa-miR-10a-3p | 7.03 | 0.009 | 0.033 |
| hsa-miR-5100 | 6.65 | <0.001 | 0.001 |
| hsa-miR-151b | 6.34 | <0.001 | <0.001 |
| hsa-miR-3138 | 6.15 | <0.001 | <0.001 |
| hsa-miR-574-3p | 5.92 | 0.002 | 0.007 |
| hsa-miR-548d-5p | 5.72 | <0.001 | 0.001 |
| hsa-miR-3614-5p | 5.64 | 0.002 | 0.010 |
| hsa-miR-6734-5p | 5.59 | <0.001 | 0.002 |
| hsa-miR-23a-5p | 5.26 | 0.008 | 0.030 |
| hsa-miR-505-5p | 5.00 | <0.001 | <0.001 |
| hsa-miR-766-5p | 4.98 | <0.001 | 0.003 |
| hsa-miR-320d | 4.98 | <0.001 | <0.001 |
| hsa-miR-664a-3p | 4.92 | <0.001 | <0.001 |
| hsa-miR-4429 | 4.72 | <0.001 | 0.001 |
| hsa-miR-148b-5p | 4.50 | 0.003 | 0.014 |
| hsa-miR-151a-5p | 4.42 | 0.001 | 0.006 |
| hsa-miR-27b-5p | 4.35 | 0.010 | 0.033 |
| hsa-miR-450a-2-3p | 4.27 | <0.001 | <0.001 |
| hsa-miR-320e | 4.25 | <0.001 | <0.001 |
| hsa-miR-320b | 4.24 | <0.001 | <0.001 |
| hsa-miR-629-5p | 4.20 | <0.001 | <0.001 |
| hsa-miR-1260b | 4.20 | 0.004 | 0.015 |
| hsa-miR-320c | 4.10 | <0.001 | <0.001 |
| hsa-miR-4443 | 3.77 | 0.005 | 0.020 |
| hsa-miR-760 | 3.62 | 0.001 | 0.005 |
| hsa-miR-193b-5p | 3.57 | 0.001 | 0.006 |
| hsa-miR-651-5p | 3.56 | 0.002 | 0.008 |
| hsa-miR-15a-5p | 3.56 | 0.001 | 0.004 |
| hsa-miR-26b-3p | 3.55 | 0.010 | 0.034 |
| hsa-miR-193a-5p | 3.36 | <0.001 | 0.001 |
| hsa-miR-99b-3p | 3.25 | 0.015 | 0.049 |
| hsa-miR-22-3p | 3.24 | <0.001 | <0.001 |
| hsa-miR-191-3p | 3.15 | 0.006 | 0.023 |
| hsa-miR-92b-3p | 3.05 | <0.001 | <0.001 |
| hsa-miR-1908-5p | 2.99 | 0.001 | 0.003 |
| hsa-miR-125b-5p | 2.98 | <0.001 | <0.001 |
| hsa-miR-502-3p | 2.89 | <0.001 | 0.002 |
| hsa-miR-22-5p | 2.88 | <0.001 | <0.001 |
| hsa-miR-425-3p | 2.86 | <0.001 | 0.003 |
| hsa-miR-503-5p | 2.77 | 0.001 | 0.007 |
| hsa-miR-320a-3p | 2.76 | 0.003 | 0.013 |
| hsa-miR-107 | 2.76 | <0.001 | <0.001 |
| hsa-let-7b-3p | 2.72 | 0.005 | 0.020 |
| hsa-miR-576-3p | 2.63 | 0.004 | 0.017 |
| hsa-miR-378c | 2.56 | 0.010 | 0.034 |
| hsa-miR-1273h-3p | 2.46 | 0.011 | 0.036 |
| hsa-miR-194-5p | 2.44 | <0.001 | 0.002 |
| hsa-miR-5010-3p | 2.43 | <0.001 | <0.001 |
| hsa-miR-1246 | 2.43 | 0.002 | 0.008 |
| hsa-miR-1290 | 2.35 | 0.005 | 0.019 |
| hsa-miR-2115-3p | 2.31 | <0.001 | <0.001 |
| hsa-miR-501-3p | 2.30 | <0.001 | 0.002 |
| hsa-miR-25-5p | 2.26 | 0.009 | 0.033 |
| hsa-miR-1301-3p | 2.25 | 0.010 | 0.034 |
| hsa-miR-744-5p | 2.08 | 0.002 | 0.010 |
| hsa-miR-126-5p | 2.04 | 0.008 | 0.030 |
| hsa-miR-4732-3p | 2.01 | 0.010 | 0.034 |
| hsa-miR-99b-5p | 1.94 | <0.001 | 0.001 |
| hsa-miR-185-5p | 1.90 | 0.005 | 0.019 |
| hsa-miR-103a-3p | 1.84 | <0.001 | <0.001 |
| hsa-let-7d-3p | 1.84 | 0.002 | 0.008 |
| hsa-let-7a-3p | 1.82 | <0.001 | <0.001 |
| hsa-miR-1843 | 1.81 | 0.004 | 0.018 |
| hsa-miR-423-5p | 1.79 | 0.004 | 0.015 |
| hsa-miR-181b-5p | 1.77 | 0.002 | 0.010 |
| hsa-miR-27b-3p | 1.77 | 0.002 | 0.009 |
| hsa-miR-486-5p | 1.74 | 0.006 | 0.024 |
| hsa-let-7d-5p | 1.70 | 0.003 | 0.011 |
| hsa-miR-16-2-3p | 1.65 | 0.004 | 0.017 |
| hsa-miR-423-3p | 1.59 | <0.001 | 0.003 |
| hsa-miR-92a-3p | 1.58 | 0.002 | 0.008 |
| hsa-miR-7-5p | 1.55 | 0.015 | 0.048 |
| hsa-miR-21-5p | 1.53 | <0.001 | <0.001 |
| hsa-miR-501-5p | 0.66 | <0.001 | 0.001 |
| hsa-miR-186-5p | 0.64 | 0.002 | 0.008 |
| hsa-miR-532-5p | 0.64 | 0.007 | 0.024 |
| hsa-miR-708-3p | 0.63 | <0.001 | <0.001 |
| hsa-let-7g-5p | 0.62 | <0.001 | <0.001 |
| hsa-miR-4533 | 0.60 | <0.001 | <0.001 |
| hsa-miR-328-3p | 0.57 | 0.006 | 0.022 |
| hsa-miR-93-3p | 0.56 | <0.001 | <0.001 |
| hsa-miR-6805-5p | 0.56 | <0.001 | <0.001 |
| hsa-miR-139-5p | 0.55 | <0.001 | 0.003 |
| hsa-miR-532-3p | 0.55 | <0.001 | <0.001 |
| hsa-miR-374b-5p | 0.54 | <0.001 | <0.001 |
| hsa-miR-106b-5p | 0.54 | 0.014 | 0.046 |
| hsa-miR-487a-5p | 0.54 | <0.001 | <0.001 |
| hsa-miR-151a-3p | 0.53 | <0.001 | 0.001 |
| hsa-miR-409-5p | 0.52 | <0.001 | <0.001 |
| hsa-miR-671-3p | 0.48 | 0.007 | 0.027 |
| hsa-miR-125b-1-3p | 0.47 | <0.001 | <0.001 |
| hsa-miR-329-3p | 0.47 | <0.001 | <0.001 |
| hsa-miR-7705 | 0.46 | <0.001 | <0.001 |
| hsa-miR-215-5p | 0.44 | 0.002 | 0.008 |
| hsa-miR-4435 | 0.43 | <0.001 | <0.001 |
| hsa-miR-487b-3p | 0.42 | <0.001 | <0.001 |
| hsa-miR-548j-3p | 0.42 | <0.001 | <0.001 |
| hsa-miR-340-3p | 0.40 | 0.001 | 0.007 |
| hsa-miR-155-5p | 0.37 | <0.001 | 0.001 |
| hsa-miR-4746-5p | 0.35 | <0.001 | <0.001 |
| hsa-miR-30b-5p | 0.33 | 0.015 | 0.050 |
| hsa-miR-1228-5p | 0.30 | 0.004 | 0.018 |
| hsa-miR-4516 | 0.28 | 0.003 | 0.015 |
| hsa-miR-342-3p | 0.26 | 0.003 | 0.014 |
| hsa-miR-4433b-3p | 0.25 | <0.001 | 0.001 |
| hsa-miR-146b-5p | 0.25 | <0.001 | <0.001 |
| hsa-miR-195-5p | 0.24 | 0.006 | 0.022 |
| hsa-miR-27a-5p | 0.24 | 0.004 | 0.016 |
| hsa-miR-7848-3p | 0.22 | <0.001 | <0.001 |
| hsa-miR-132-5p | 0.20 | 0.001 | 0.003 |
| hsa-miR-3916 | 0.20 | 0.004 | 0.016 |
| hsa-miR-26a-1-3p | 0.18 | <0.001 | <0.001 |
| hsa-miR-150-3p | 0.18 | <0.001 | <0.001 |
| hsa-miR-642a-3p | 0.16 | 0.004 | 0.017 |
| hsa-miR-342-5p | 0.15 | <0.001 | <0.001 |
| hsa-miR-150-5p | 0.13 | <0.001 | <0.001 |
| hsa-miR-184 | 0.13 | 0.004 | 0.016 |
| hsa-miR-3690 | 0.11 | 0.007 | 0.026 |
| hsa-miR-3158-3p | 0.11 | <0.001 | <0.001 |
| hsa-miR-6754-5p | 0.10 | 0.005 | 0.020 |
| hsa-miR-296-3p | 0.10 | 0.001 | 0.007 |
| hsa-miR-2115-5p | 0.08 | 0.012 | 0.039 |
| hsa-miR-6869-5p | 0.07 | 0.007 | 0.026 |
| hsa-miR-4433a-3p | 0.07 | <0.001 | <0.001 |
| hsa-miR-516b-5p | 0.06 | 0.013 | 0.045 |
| hsa-miR-3934-5p | 0.06 | 0.013 | 0.045 |
| hsa-miR-659-5p | 0.06 | <0.001 | 0.002 |
| hsa-miR-619-5p | 0.04 | 0.001 | 0.004 |
| hsa-miR-1275 | 0.03 | <0.001 | <0.001 |
| hsa-miR-3165 | 0.03 | 0.006 | 0.022 |
| hsa-miR-574-5p | 0.03 | <0.001 | <0.001 |

## **Supplementary Data 7: miRNA network of COVID+.**

MiRNA– targeted genes interaction with significantly enriched pathways for SDE miRNAs between COVID+ patients and healthy controls. Statistically significant experimentally validated miRNA-gene target interactions were obtained with miRTarbase (p-value <0.05 adjusted by FDR using Benjamin-Hochberg correction and a minimum of two interactions). The g:Profiler was used to conduct a functional enrichment analysis on the targeted enriched genes and the KEGG (Kyoto Encyclopaedia of Genes and Genomes) database to retrieve statistically significantly enriched pathways (adjusted p-value <0.05).

**
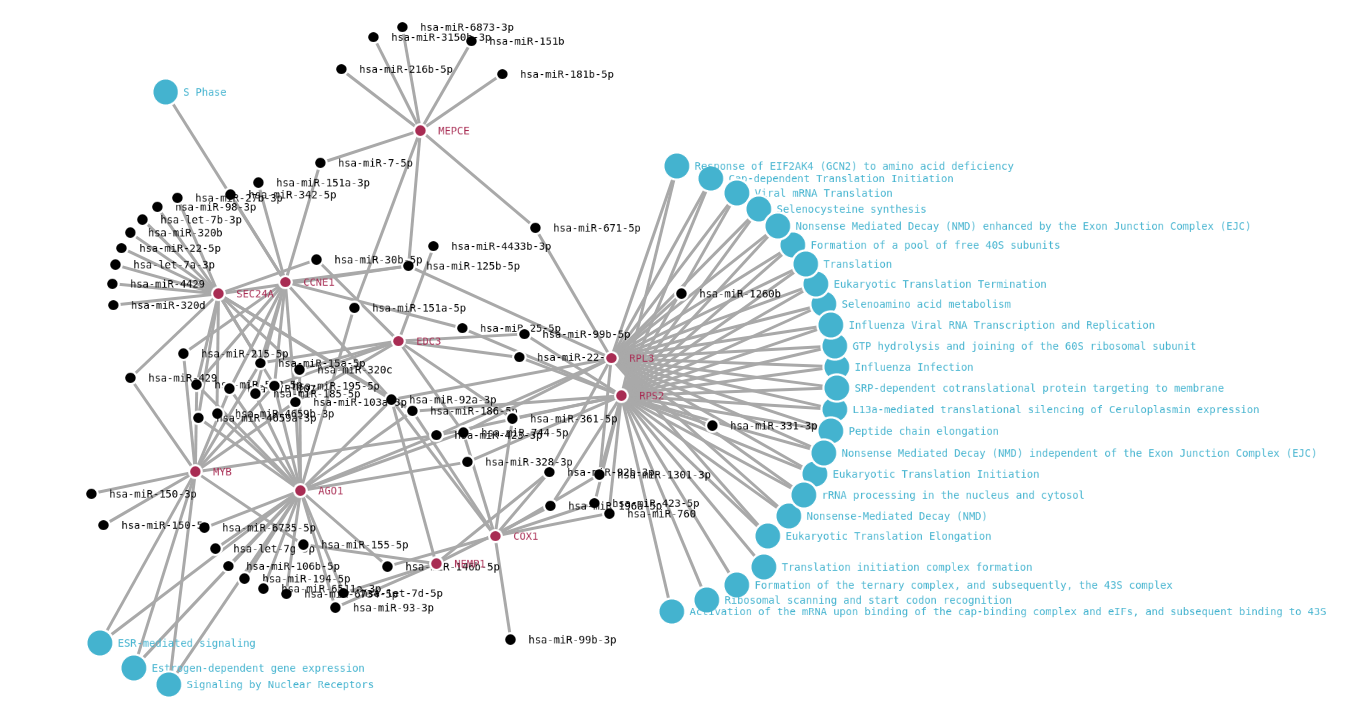
**

## **Supplementary Data 8:** miRNA-targeted pathways for significant differentially expressed (SDE) miRNAs of COVID+ patient’s vs. healthy controls.

| **Pathway ID** | **Description** | **Gene Ratio** | **Bg Ratio** | **p-value** | **p-adjust** | **gene ID** | **Count** |
| --- | --- | --- | --- | --- | --- | --- | --- |
| R-HSA-9018519 | Estrogen-dependent gene expression | 6/54 | 150/10704 | <0.001 | 0.01 | AGO1/BCL2/MYB/MYC/POLR2L/RAD21 | 6 |
| R-HSA-8939211 | ESR-mediated signaling | 7/54 | 223/10704 | <0.001 | 0.01 | AGO1/BCL2/IGF1R/MYB/MYC/POLR2L/RAD21 | 7 |
| R-HSA-168255 | Influenza Infection | 6/54 | 156/10704 | <0.001 | 0.01 | HSPA1B/POLR2L/RPL11/RPL3/RPS2/RPS3A | 6 |
| R-HSA-72689 | Formation of a pool of free 40S subunits | 5/54 | 101/10704 | <0.001 | 0.01 | EIF3C/RPL11/RPL3/RPS2/RPS3A | 5 |
| R-HSA-69242 | S Phase | 6/54 | 162/10704 | <0.001 | 0.01 | CCNE1/GSK3B/MYC/POLD3/POLE4/RAD21 | 6 |
| R-HSA-156827 | L13a-mediated translational silencing of Ceruloplasmin expression | 5/54 | 111/10704 | <0.001 | 0.01 | EIF3C/RPL11/RPL3/RPS2/RPS3A | 5 |
| R-HSA-72706 | GTP hydrolysis and joining of the 60S ribosomal subunit | 5/54 | 112/10704 | <0.001 | 0.01 | EIF3C/RPL11/RPL3/RPS2/RPS3A | 5 |
| R-HSA-2408522 | Selenoamino acid metabolism | 5/54 | 118/10704 | <0.001 | 0.01 | AIMP1/RPL11/RPL3/RPS2/RPS3A | 5 |
| R-HSA-72613 | Eukaryotic Translation Initiation | 5/54 | 119/10704 | <0.001 | 0.01 | EIF3C/RPL11/RPL3/RPS2/RPS3A | 5 |
| R-HSA-72737 | Cap-dependent Translation Initiation | 5/54 | 119/10704 | <0.001 | 0.01 | EIF3C/RPL11/RPL3/RPS2/RPS3A | 5 |
| R-HSA-168273 | Influenza Viral RNA Transcription and Replication | 5/54 | 135/10704 | 0.001 | 0.02 | POLR2L/RPL11/RPL3/RPS2/RPS3A | 5 |
| R-HSA-9006931 | Signaling by Nuclear Receptors | 7/54 | 299/10704 | 0.001 | 0.03 | AGO1/BCL2/IGF1R/MYB/MYC/POLR2L/RAD21 | 7 |
| R-HSA-8864260 | Transcriptional regulation by the AP-2 (TFAP2) family of transcription factors | 3/54 | 38/10704 | 0.001 | 0.03 | MYC/NOP2/TFAP2A | 3 |
| R-HSA-156902 | Peptide chain elongation | 4/54 | 89/10704 | 0.001 | 0.03 | RPL11/RPL3/RPS2/RPS3A | 4 |
| R-HSA-192823 | Viral mRNA Translation | 4/54 | 89/10704 | 0.001 | 0.03 | RPL11/RPL3/RPS2/RPS3A | 4 |
| R-HSA-156842 | Eukaryotic Translation Elongation | 4/54 | 93/10704 | 0.001 | 0.03 | RPL11/RPL3/RPS2/RPS3A | 4 |
| R-HSA-2408557 | Selenocysteine synthesis | 4/54 | 93/10704 | 0.001 | 0.03 | RPL11/RPL3/RPS2/RPS3A | 4 |
| R-HSA-72764 | Eukaryotic Translation Termination | 4/54 | 93/10704 | 0.001 | 0.03 | RPL11/RPL3/RPS2/RPS3A | 4 |
| R-HSA-975956 | Nonsense Mediated Decay (NMD) independent of the Exon Junction Complex (EJC) | 4/54 | 95/10704 | 0.001 | 0.03 | RPL11/RPL3/RPS2/RPS3A | 4 |
| R-HSA-9633012 | Response of EIF2AK4 (GCN2) to amino acid deficiency | 4/54 | 101/10704 | 0.002 | 0.04 | RPL11/RPL3/RPS2/RPS3A | 4 |
| R-HSA-72695 | Formation of the ternary complex. and subsequently. the 43S complex | 3/54 | 51/10704 | 0.002 | 0.04 | EIF3C/RPS2/RPS3A | 3 |
| R-HSA-1799339 | SRP-dependent cotranslational protein targeting to membrane | 4/54 | 112/10704 | 0.002 | 0.05 | RPL11/RPL3/RPS2/RPS3A | 4 |
| R-HSA-927802 | Nonsense-Mediated Decay (NMD) | 4/54 | 115/10704 | 0.003 | 0.05 | RPL11/RPL3/RPS2/RPS3A | 4 |
| R-HSA-975957 | Nonsense Mediated Decay (NMD) enhanced by the Exon Junction Complex (EJC) | 4/54 | 115/10704 | 0.003 | 0.05 | RPL11/RPL3/RPS2/RPS3A | 4 |
| R-HSA-8868773 | rRNA processing in the nucleus and cytosol | 5/54 | 194/10704 | 0.003 | 0.05 | NOP2/RPL11/RPL3/RPS2/RPS3A | 5 |
| R-HSA-72649 | Translation initiation complex formation | 3/54 | 58/10704 | 0.003 | 0.05 | EIF3C/RPS2/RPS3A | 3 |
| R-HSA-72702 | Ribosomal scanning and start codon recognition | 3/54 | 58/10704 | 0.003 | 0.05 | EIF3C/RPS2/RPS3A | 3 |
| R-HSA-72662 | Activation of the mRNA upon binding of the cap-binding complex and eIFs. and subsequent binding to 43S | 3/54 | 59/10704 | 0.003 | 0.05 | EIF3C/RPS2/RPS3A | 3 |
| R-HSA-72766 | Translation | 6/54 | 291/10704 | 0.003 | 0.05 | AIMP1/EIF3C/RPL11/RPL3/RPS2/RPS3A | 6 |

## **
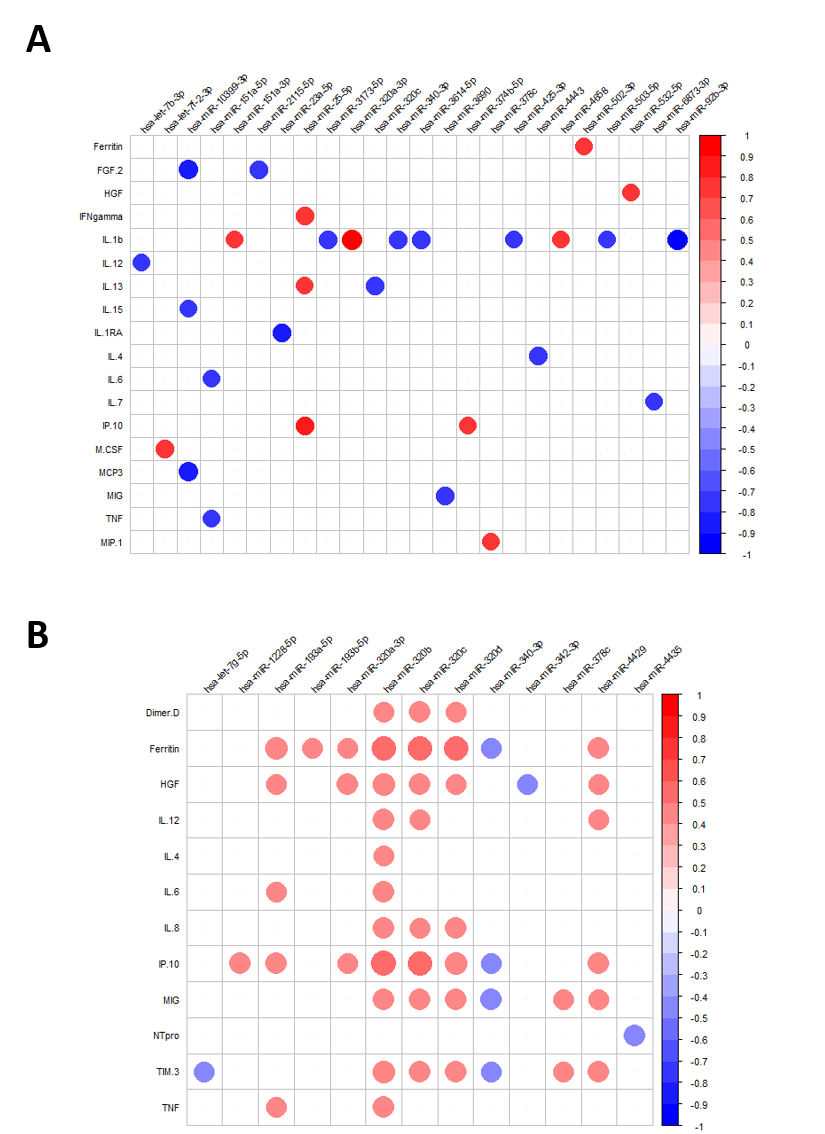
Supplementary Data 9:** Spearman correlation plot showing the correlation between SDE miRNAs and plasma cytokines / chemokines for: A) healthy controls and B) COVID+ patients. The size of the circles is proportional to the strength of the correlation and the colour represents the direction (colour legends is shown on the right), where large dark blue represents a strong negative correlation and a large dark red circle represents a strong positive correlation. SDE miRNAs are on the horizontal axis and cytokines / chemokines on the vertical axis.

## **Supplementary Data 10**: Significant differentially expressed (SDE) miRNAs between asymptomatic and symptomatic COVID+ patients (absolute fold change (FC) ≥ 1.5 and FDR ≤ 0.05).

| **MiRNA** | **FC** | **p-value** | **FDR** |
| --- | --- | --- | --- |
| hsa-miR-1291 | 14.601 | 0.006 | 0.040 |
| hsa-miR-150-5p | 4.767 | <0.001 | <0.001 |
| hsa-miR-371b-5p | 3.713 | <0.001 | <0.001 |
| hsa-miR-708-3p | 2.652 | <0.001 | <0.001 |
| hsa-miR-375-3p | 2.438 | 0.006 | 0.044 |
| hsa-miR-10b-5p | 2.345 | <0.001 | 0.003 |
| hsa-miR-410-3p | 2.336 | <0.001 | <0.001 |
| hsa-miR-382-3p | 2.202 | <0.001 | <0.001 |
| hsa-miR-32-3p | 2.168 | <0.001 | <0.001 |
| hsa-miR-4732-5p | 2.128 | 0.004 | 0.029 |
| hsa-miR-4662a-5p | 2.079 | <0.001 | <0.001 |
| hsa-miR-10a-5p | 2.041 | <0.001 | <0.001 |
| hsa-miR-6818-3p | 1.970 | <0.001 | <0.001 |
| hsa-miR-202-5p | 1.916 | <0.001 | <0.001 |
| hsa-miR-486-5p | 1.806 | 0.006 | 0.041 |
| hsa-miR-2276-3p | 1.795 | <0.001 | <0.001 |
| hsa-miR-6808-3p | 1.607 | 0.001 | 0.007 |
| hsa-miR-139-5p | 1.604 | 0.006 | 0.044 |
| hsa-miR-6513-3p | 1.512 | <0.001 | <0.001 |
| hsa-miR-5009-5p | 0.650 | <0.001 | 0.001 |
| hsa-miR-186-5p | 0.647 | 0.004 | 0.031 |
| hsa-miR-18a-5p | 0.636 | <0.001 | <0.001 |
| hsa-miR-548ag | 0.631 | <0.001 | <0.001 |
| hsa-miR-548l | 0.613 | 0.001 | 0.011 |
| hsa-miR-380-5p | 0.601 | 0.001 | 0.007 |
| hsa-miR-450a-2-3p | 0.577 | 0.001 | 0.011 |
| hsa-miR-222-3p | 0.553 | <0.001 | 0.002 |
| hsa-miR-4645-3p | 0.545 | <0.001 | 0.003 |
| hsa-miR-6716-3p | 0.544 | <0.001 | <0.001 |
| hsa-miR-378a-5p | 0.533 | <0.001 | <0.001 |
| hsa-miR-5010-3p | 0.529 | <0.001 | <0.001 |
| hsa-miR-941 | 0.516 | 0.004 | 0.033 |
| hsa-miR-552-3p | 0.504 | <0.001 | <0.001 |
| hsa-miR-505-3p | 0.497 | <0.001 | <0.001 |
| hsa-miR-664b-5p | 0.495 | <0.001 | <0.001 |
| hsa-miR-605-3p | 0.492 | <0.001 | <0.001 |
| hsa-miR-106b-3p | 0.483 | <0.001 | <0.001 |
| hsa-miR-125b-1-3p | 0.466 | <0.001 | <0.001 |
| hsa-miR-127-3p | 0.458 | 0.007 | 0.050 |
| hsa-miR-19b-3p | 0.447 | 0.001 | 0.011 |
| hsa-miR-6783-3p | 0.430 | <0.001 | <0.001 |
| hsa-miR-4435 | 0.418 | <0.001 | <0.001 |
| hsa-miR-330-3p | 0.414 | 0.004 | 0.033 |
| hsa-miR-597-3p | 0.395 | <0.001 | <0.001 |
| hsa-miR-1301-3p | 0.387 | 0.001 | 0.013 |
| hsa-miR-1273h-3p | 0.372 | 0.004 | 0.034 |
| hsa-miR-6819-3p | 0.357 | 0.002 | 0.019 |
| hsa-miR-320c | 0.356 | 0.004 | 0.032 |
| hsa-miR-6514-3p | 0.354 | <0.001 | 0.002 |
| hsa-miR-628-5p | 0.353 | <0.001 | <0.001 |
| hsa-miR-6850-5p | 0.352 | <0.001 | <0.001 |
| hsa-miR-548k | 0.352 | <0.001 | <0.001 |
| hsa-miR-30b-3p | 0.352 | <0.001 | <0.001 |
| hsa-miR-504-5p | 0.347 | <0.001 | <0.001 |
| hsa-miR-1307-3p | 0.312 | <0.001 | <0.001 |
| hsa-miR-193a-5p | 0.308 | <0.001 | 0.005 |
| hsa-miR-320b | 0.302 | 0.001 | 0.011 |
| hsa-miR-10399-3p | 0.293 | 0.003 | 0.027 |
| hsa-miR-1908-5p | 0.288 | <0.001 | 0.001 |
| hsa-miR-6852-5p | 0.254 | 0.004 | 0.033 |
| hsa-miR-625-5p | 0.242 | <0.001 | <0.001 |
| hsa-miR-7854-3p | 0.216 | 0.003 | 0.026 |
| hsa-miR-370-3p | 0.205 | <0.001 | 0.001 |
| hsa-miR-200b-3p | 0.199 | <0.001 | 0.003 |
| hsa-miR-1228-5p | 0.188 | <0.001 | 0.002 |
| hsa-miR-6891-5p | 0.114 | <0.001 | 0.004 |
| hsa-miR-424-5p | 0.095 | 0.003 | 0.026 |
| hsa-miR-939-5p | 0.083 | <0.001 | 0.002 |
| hsa-miR-26a-2-3p | 0.074 | 0.002 | 0.020 |
| hsa-miR-296-5p | 0.069 | <0.001 | <0.001 |
| hsa-miR-4750-5p | 0.068 | 0.006 | 0.040 |
| hsa-miR-937-3p | 0.057 | <0.001 | 0.002 |
| hsa-miR-412-5p | 0.023 | 0.005 | 0.037 |
| hsa-miR-1229-3p | 0.012 | 0.001 | 0.006 |
| hsa-miR-8485 | 0.010 | 0.001 | 0.010 |

## **Supplementary Data 11: miRNA network of COVID+ asymptomatic patients**.

MiRNA– targeted genes interaction with significantly enriched pathways for SDE miRNAs between asymptomatic (AM) and symtomatic patients. Statistically significant experimentally validated miRNA-gene target interactions were obtained with miRTarbase (q-value <0.05 adjusted by false discovery rate (FDR) using Benjamin-Hochberg correction and a minimum of two interactions). The g:Profiler was used to conduct a functional enrichment analysis on the targeted enriched genes and the KEGG (Kyoto Encyclopaedia of Genes and Genomes) database to retrieve statistically significantly enriched pathways (adjusted p-value <0.05).

**
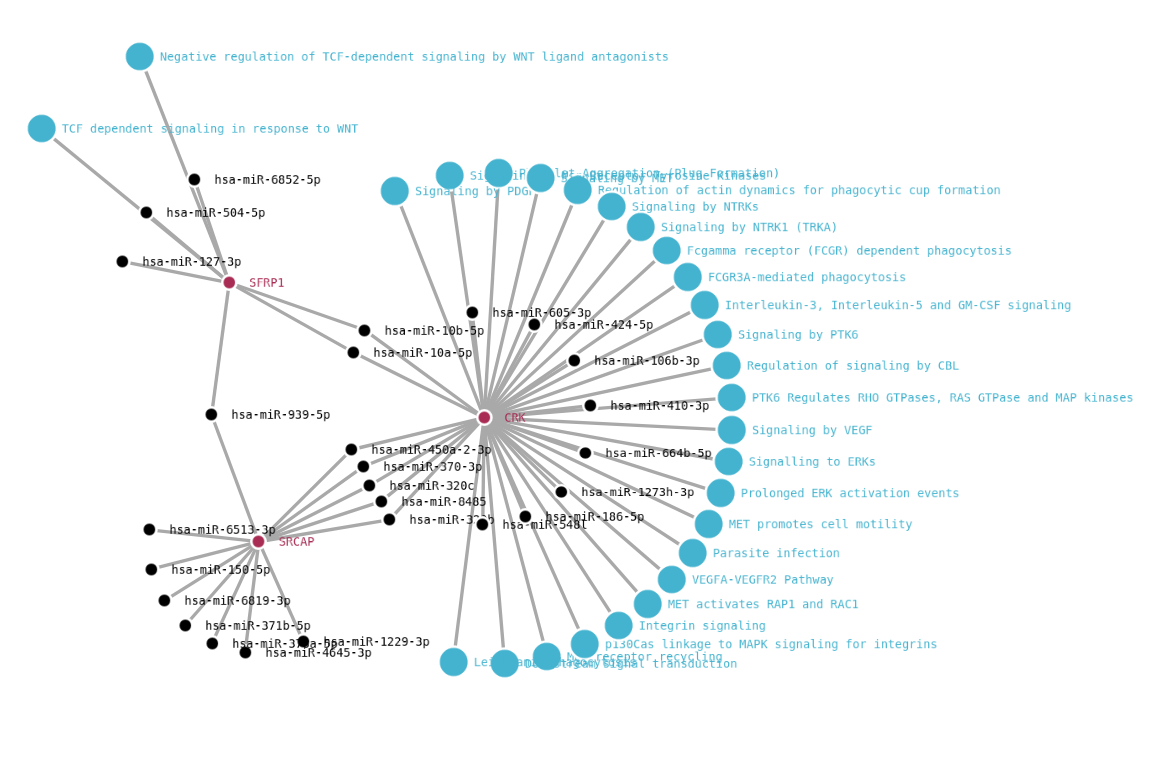
**

## **Supplementary Data 12:** miRNA-targeted pathways for SDE miRNAs of asymptomatic vs. symptomatic patients.

| **Pathway ID** | **Description** | **Gene ratio** | **Bg ratio** | **p-value** | **p-adjust** | **gene ID** | **Count** |
| --- | --- | --- | --- | --- | --- | --- | --- |
| R-HSA-8875656 | MET receptor recycling | 1/2 | 10/10704 | 0.002 | 0.014 | CRK | 1 |
| R-HSA-8875555 | MET activates RAP1 and RAC1 | 1/2 | 11/10704 | 0.002 | 0.014 | CRK | 1 |
| R-HSA-169893 | Prolonged ERK activation events | 1/2 | 14/10704 | 0.003 | 0.014 | CRK | 1 |
| R-HSA-8849471 | PTK6 Regulates RHO GTPases. RAS GTPase and MAP kinases | 1/2 | 14/10704 | 0.003 | 0.014 | CRK | 1 |
| R-HSA-372708 | p130Cas linkage to MAPK signaling for integrins | 1/2 | 15/10704 | 0.003 | 0.014 | CRK | 1 |
| R-HSA-3772470 | Negative regulation of TCF-dependent signaling by WNT ligand antagonists | 1/2 | 15/10704 | 0.003 | 0.014 | SFRP1 | 1 |
| R-HSA-912631 | Regulation of signaling by CBL | 1/2 | 22/10704 | 0.004 | 0.018 | CRK | 1 |
| R-HSA-354192 | Integrin signaling | 1/2 | 27/10704 | 0.005 | 0.018 | CRK | 1 |
| R-HSA-186763 | Downstream signal transduction | 1/2 | 29/10704 | 0.005 | 0.018 | CRK | 1 |
| R-HSA-187687 | Signalling to ERKs | 1/2 | 34/10704 | 0.006 | 0.018 | CRK | 1 |
| R-HSA-76009 | Platelet Aggregation (Plug Formation) | 1/2 | 39/10704 | 0.007 | 0.018 | CRK | 1 |
| R-HSA-8875878 | MET promotes cell motility | 1/2 | 41/10704 | 0.008 | 0.018 | CRK | 1 |
| R-HSA-512988 | Interleukin-3. Interleukin-5 and GM-CSF signaling | 1/2 | 48/10704 | 0.009 | 0.018 | CRK | 1 |
| R-HSA-8848021 | Signaling by PTK6 | 1/2 | 54/10704 | 0.010 | 0.018 | CRK | 1 |
| R-HSA-9006927 | Signaling by Non-Receptor Tyrosine Kinases | 1/2 | 54/10704 | 0.010 | 0.018 | CRK | 1 |
| R-HSA-186797 | Signaling by PDGF | 1/2 | 58/10704 | 0.011 | 0.018 | CRK | 1 |
| R-HSA-9664407 | Parasite infection | 1/2 | 59/10704 | 0.011 | 0.018 | CRK | 1 |
| R-HSA-9664417 | Leishmania phagocytosis | 1/2 | 59/10704 | 0.011 | 0.018 | CRK | 1 |
| R-HSA-9664422 | FCGR3A-mediated phagocytosis | 1/2 | 59/10704 | 0.011 | 0.018 | CRK | 1 |
| R-HSA-2029482 | Regulation of actin dynamics for phagocytic cup formation | 1/2 | 61/10704 | 0.011 | 0.018 | CRK | 1 |
| R-HSA-6806834 | Signaling by MET | 1/2 | 79/10704 | 0.015 | 0.022 | CRK | 1 |
| R-HSA-2029480 | Fcgamma receptor (FCGR) dependent phagocytosis | 1/2 | 86/10704 | 0.016 | 0.023 | CRK | 1 |
| R-HSA-4420097 | VEGFA-VEGFR2 Pathway | 1/2 | 99/10704 | 0.018 | 0.025 | CRK | 1 |
| R-HSA-194138 | Signaling by VEGF | 1/2 | 108/10704 | 0.020 | 0.026 | CRK | 1 |
| R-HSA-187037 | Signaling by NTRK1 (TRKA) | 1/2 | 115/10704 | 0.021 | 0.027 | CRK | 1 |
| R-HSA-166520 | Signaling by NTRKs | 1/2 | 134/10704 | 0.025 | 0.030 | CRK | 1 |
| R-HSA-201681 | TCF dependent signalling in response to WNT | 1/2 | 233/10704 | 0.043 | 0.049 | SFRP1 | 1 |

## **Supplementary Data 13:** Pearson correlation plot showing the correlation between SDE miRNAs and plasma cytokines / chemokines for: A) Asymtomatic and B) Symptomatic. The size of the circles is proportional to the strength of the correlation and the colour represents the direction (colour legends is shown on the right), where large dark blue represents a strong negative correlation and a large dark red circle represents a strong positive correlation. SDE miRNAs are on the horizontal axis and cytokines / chemokines on the vertical axis.

**
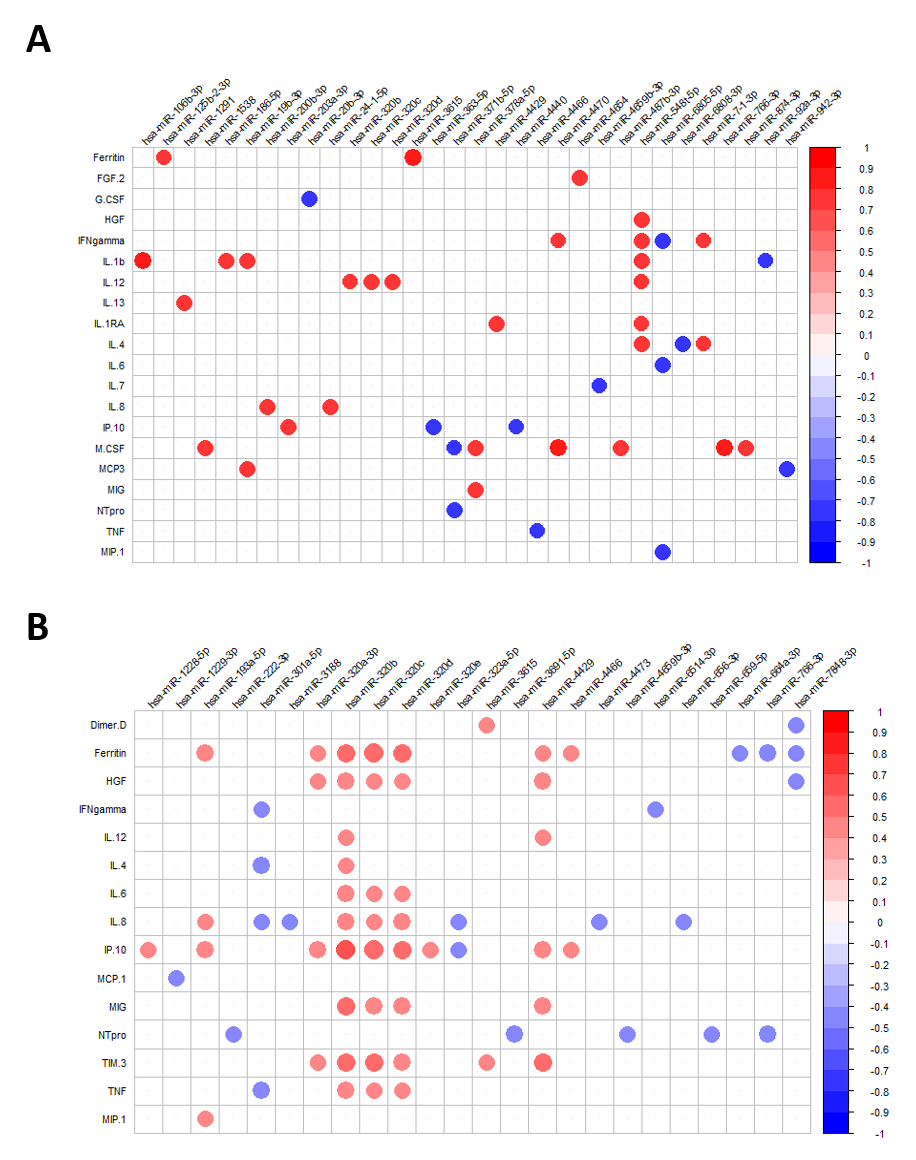
**

## **Supplementary Data 14**: Significant differentially expressed (SDE) miRNAs between severe and moderate COVID+ patients (absolute fold change (FC) ≥ 1.5 and FDR ≤ 0.05).

| **MiRNA** | **FC** | **p-value** | **FDR** |
| --- | --- | --- | --- |
| hsa-miR-3976 | 33.50 | <0.001 | <0.001 |
| hsa-miR-4488 | 20.61 | <0.001 | <0.001 |
| hsa-miR-3150b-3p | 16.62 | 0.003 | 0.019 |
| hsa-miR-7704 | 13.61 | <0.001 | 0.003 |
| hsa-miR-3168 | 10.80 | <0.001 | <0.001 |
| hsa-miR-3960 | 9.93 | <0.001 | <0.001 |
| hsa-miR-7977 | 9.14 | <0.001 | 0.002 |
| hsa-miR-8485 | 8.46 | 0.008 | 0.038 |
| hsa-miR-1260a | 8.00 | <0.001 | <0.001 |
| hsa-miR-320d | 6.75 | <0.001 | <0.001 |
| hsa-miR-4429 | 6.29 | <0.001 | <0.001 |
| hsa-miR-320a-3p | 6.15 | <0.001 | <0.001 |
| hsa-miR-320b | 6.03 | <0.001 | <0.001 |
| hsa-miR-937-3p | 6.03 | <0.001 | 0.004 |
| hsa-miR-320e | 5.97 | <0.001 | <0.001 |
| hsa-miR-203a-3p | 5.71 | <0.001 | <0.001 |
| hsa-miR-320c | 5.69 | <0.001 | <0.001 |
| hsa-miR-4508 | 4.96 | <0.001 | <0.001 |
| hsa-miR-1287-5p | 4.90 | <0.001 | 0.004 |
| hsa-miR-939-5p | 4.73 | 0.001 | 0.009 |
| hsa-miR-7854-3p | 4.47 | <0.001 | <0.001 |
| hsa-miR-1260b | 4.34 | <0.001 | <0.001 |
| hsa-miR-1228-5p | 4.29 | <0.001 | <0.001 |
| hsa-miR-193a-5p | 4.26 | <0.001 | <0.001 |
| hsa-miR-181a-3p | 4.17 | <0.001 | 0.002 |
| hsa-miR-184 | 4.06 | 0.005 | 0.026 |
| hsa-miR-150-3p | 4.04 | <0.001 | <0.001 |
| hsa-miR-548h-3p | 3.54 | 0.011 | 0.050 |
| hsa-miR-548z | 3.54 | 0.011 | 0.050 |
| hsa-miR-193b-3p | 3.50 | <0.001 | <0.001 |
| hsa-miR-5100 | 3.39 | 0.001 | 0.005 |
| hsa-miR-23a-5p | 3.37 | 0.006 | 0.031 |
| hsa-miR-576-3p | 3.31 | <0.001 | <0.001 |
| hsa-miR-6837-3p | 3.30 | <0.001 | <0.001 |
| hsa-miR-433-3p | 3.28 | 0.004 | 0.025 |
| hsa-miR-5588-5p | 3.25 | <0.001 | <0.001 |
| hsa-miR-378c | 3.24 | <0.001 | <0.001 |
| hsa-miR-4665-5p | 3.22 | 0.010 | 0.049 |
| hsa-miR-7847-3p | 3.20 | <0.001 | 0.004 |
| hsa-miR-134-5p | 3.18 | <0.001 | <0.001 |
| hsa-miR-483-5p | 3.07 | <0.001 | <0.001 |
| hsa-miR-4516 | 3.00 | <0.001 | 0.001 |
| hsa-miR-370-3p | 2.93 | <0.001 | 0.001 |
| hsa-miR-193b-5p | 2.89 | <0.001 | 0.003 |
| hsa-miR-1292-5p | 2.88 | 0.001 | 0.008 |
| hsa-miR-574-5p | 2.86 | 0.004 | 0.022 |
| hsa-miR-200b-5p | 2.76 | <0.001 | <0.001 |
| hsa-miR-200b-3p | 2.65 | 0.001 | 0.009 |
| hsa-miR-130a-3p | 2.63 | 0.001 | 0.009 |
| hsa-miR-330-5p | 2.50 | 0.006 | 0.031 |
| hsa-miR-7706 | 2.40 | <0.001 | 0.001 |
| hsa-miR-760 | 2.37 | 0.002 | 0.011 |
| hsa-miR-6131 | 2.35 | 0.002 | 0.011 |
| hsa-miR-1306-3p | 2.20 | 0.002 | 0.011 |
| hsa-miR-1290 | 2.17 | <0.001 | 0.004 |
| hsa-miR-425-3p | 2.05 | <0.001 | 0.002 |
| hsa-miR-1273h-3p | 2.05 | 0.004 | 0.024 |
| hsa-miR-185-5p | 2.05 | <0.001 | <0.001 |
| hsa-miR-1246 | 2.01 | 0.001 | 0.009 |
| hsa-miR-1908-5p | 2.00 | 0.003 | 0.016 |
| hsa-miR-511-5p | 1.98 | 0.007 | 0.038 |
| hsa-miR-423-5p | 1.98 | <0.001 | <0.001 |
| hsa-miR-92b-5p | 1.98 | 0.006 | 0.031 |
| hsa-miR-1307-3p | 1.97 | <0.001 | 0.001 |
| hsa-miR-150-5p | 1.97 | 0.004 | 0.025 |
| hsa-miR-375-3p | 1.95 | 0.004 | 0.024 |
| hsa-miR-337-5p | 1.88 | <0.001 | <0.001 |
| hsa-miR-629-5p | 1.88 | <0.001 | 0.003 |
| hsa-let-7c-3p | 1.84 | <0.001 | <0.001 |
| hsa-miR-664a-5p | 1.78 | 0.005 | 0.026 |
| hsa-miR-501-3p | 1.74 | 0.001 | 0.007 |
| hsa-miR-378f | 1.73 | 0.006 | 0.033 |
| hsa-miR-24-2-5p | 1.71 | 0.005 | 0.028 |
| hsa-miR-3615 | 1.68 | <0.001 | 0.001 |
| hsa-miR-342-5p | 1.65 | 0.002 | 0.011 |
| hsa-miR-139-3p | 1.64 | 0.011 | 0.050 |
| hsa-miR-29a-3p | 1.59 | 0.002 | 0.012 |
| hsa-miR-7705 | 0.66 | 0.008 | 0.041 |
| hsa-miR-584-5p | 0.66 | 0.001 | 0.008 |
| hsa-miR-548k | 0.66 | <0.001 | <0.001 |
| hsa-let-7a-3p | 0.66 | <0.001 | <0.001 |
| hsa-miR-328-3p | 0.64 | 0.008 | 0.039 |
| hsa-miR-18a-5p | 0.64 | <0.001 | <0.001 |
| hsa-miR-26a-5p | 0.63 | 0.001 | 0.009 |
| hsa-miR-625-3p | 0.62 | 0.008 | 0.041 |
| hsa-miR-3913-5p | 0.62 | <0.001 | <0.001 |
| hsa-miR-942-5p | 0.61 | <0.001 | <0.001 |
| hsa-miR-1827 | 0.61 | 0.008 | 0.038 |
| hsa-miR-3120-5p | 0.61 | <0.001 | <0.001 |
| hsa-miR-4435 | 0.61 | <0.001 | <0.001 |
| hsa-miR-664a-3p | 0.60 | <0.001 | <0.001 |
| hsa-miR-126-5p | 0.59 | 0.011 | 0.050 |
| hsa-miR-98-5p | 0.58 | 0.001 | 0.007 |
| hsa-miR-4738-3p | 0.58 | <0.001 | <0.001 |
| hsa-miR-548j-3p | 0.57 | <0.001 | <0.001 |
| hsa-miR-199a-5p | 0.56 | 0.004 | 0.024 |
| hsa-miR-30e-3p | 0.55 | <0.001 | 0.003 |
| hsa-miR-3617-5p | 0.55 | <0.001 | 0.003 |
| hsa-miR-4662a-5p | 0.54 | <0.001 | <0.001 |
| hsa-miR-4639-5p | 0.53 | <0.001 | <0.001 |
| hsa-miR-3164 | 0.53 | <0.001 | <0.001 |
| hsa-miR-628-3p | 0.52 | 0.007 | 0.037 |
| hsa-miR-877-5p | 0.51 | 0.007 | 0.038 |
| hsa-miR-26a-2-3p | 0.49 | <0.001 | <0.001 |
| hsa-miR-149-5p | 0.48 | <0.001 | <0.001 |
| hsa-miR-301b-3p | 0.47 | <0.001 | <0.001 |
| hsa-miR-4657 | 0.47 | <0.001 | <0.001 |
| hsa-miR-1278 | 0.46 | <0.001 | <0.001 |
| hsa-miR-628-5p | 0.41 | <0.001 | <0.001 |
| hsa-miR-331-5p | 0.38 | 0.004 | 0.025 |
| hsa-miR-6806-3p | 0.36 | <0.001 | <0.001 |
| hsa-miR-4659b-3p | 0.36 | 0.010 | 0.049 |
| hsa-miR-6764-5p | 0.35 | <0.001 | <0.001 |
| hsa-miR-6818-3p | 0.34 | <0.001 | <0.001 |
| hsa-miR-659-5p | 0.30 | <0.001 | <0.001 |
| hsa-miR-5001-3p | 0.27 | <0.001 | <0.001 |
| hsa-miR-548az-5p | 0.24 | 0.006 | 0.031 |
| hsa-miR-550b-2-5p | 0.24 | 0.009 | 0.045 |
| hsa-miR-548ag | 0.22 | 0.002 | 0.016 |
| hsa-miR-548t-5p | 0.22 | 0.008 | 0.041 |
| hsa-miR-9903 | 0.12 | 0.008 | 0.041 |
| hsa-miR-380-5p | 0.12 | 0.005 | 0.029 |
| hsa-miR-3188 | 0.11 | 0.004 | 0.024 |
| hsa-miR-6783-3p | 0.10 | <0.001 | <0.001 |
| hsa-miR-7848-3p | 0.10 | <0.001 | 0.001 |
| hsa-miR-7110-3p | 0.09 | 0.006 | 0.033 |
| hsa-miR-1197 | 0.09 | 0.010 | 0.047 |
| hsa-miR-4686 | 0.08 | <0.001 | <0.001 |
| hsa-miR-374b-3p | 0.08 | <0.001 | 0.004 |
| hsa-miR-4473 | 0.08 | <0.001 | <0.001 |
| hsa-miR-758-3p | 0.05 | <0.001 | <0.001 |
| hsa-miR-5004-3p | 0.05 | 0.011 | 0.050 |
| hsa-miR-146a-3p | 0.05 | <0.001 | <0.001 |
| hsa-miR-329-5p | 0.05 | <0.001 | 0.002 |
| hsa-miR-744-3p | 0.04 | 0.001 | 0.005 |
| hsa-miR-1285-5p | 0.01 | <0.001 | <0.001 |
| hsa-miR-6516-3p | 0.01 | <0.001 | <0.001 |

## **Supplementary Data 15. miRNA network of COVID+ severity**: miRNA– targeted genes interaction with significantly enriched pathways for SDE miRNAs between severe and moderate patients. Statistically significant experimentally validated miRNA-gene target interactions were obtained with miRTarbase (q-value <0.05 adjusted by false discovery rate (FDR) using Benjamin-Hochberg correction and a minimum of two interactions). The g:Profiler was used to conduct a functional enrichment analysis on the targeted enriched genes and the KEGG (Kyoto Encyclopaedia of Genes and Genomes) database to retrieve statistically significantly enriched pathways (q-value <0.05).

**
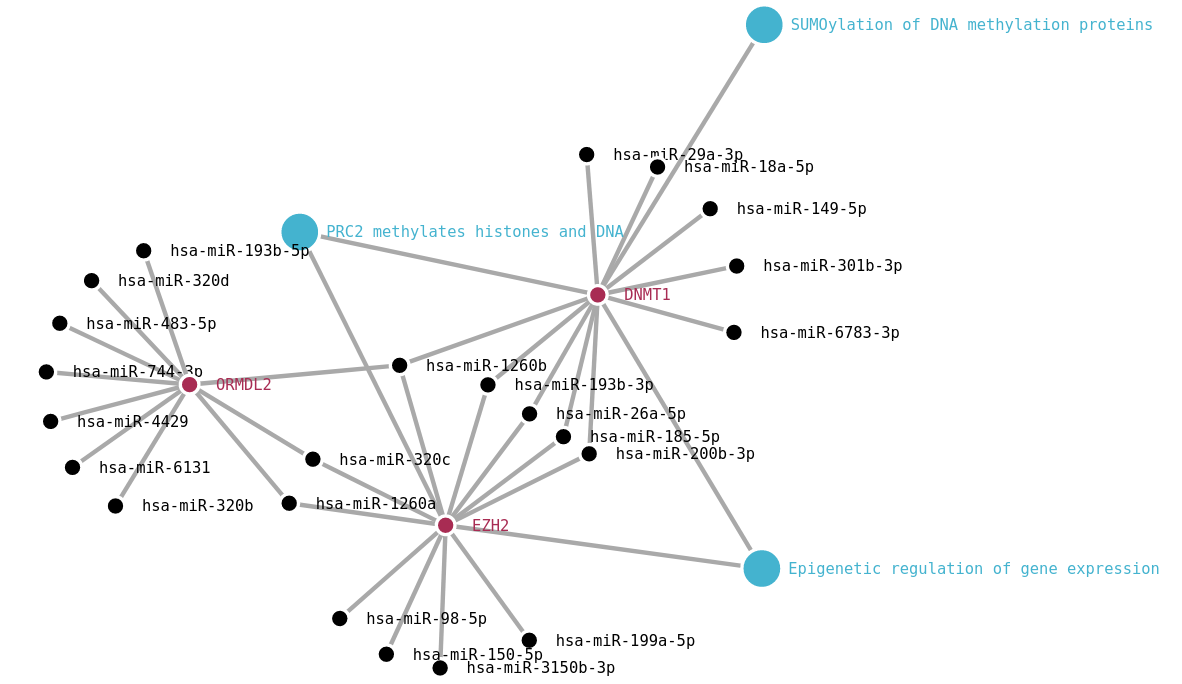
**

## **Supplementary Data 16:** miRNA-targeted pathways for significant differentially expressed (SDE) miRNAs of severe vs. moderate patients.

| **Pathway ID** | **Description** | **Gene ratio** | **Bg ratio** | **p-value** | **p-adjust** | **gene ID** | **Count** |
| --- | --- | --- | --- | --- | --- | --- | --- |
| R-HSA-212300 | PRC2 methylates histones and DNA | 2/3 | 73/10704 | <0.001 | 0.001 | DNMT1/EZH2 | 2 |
| R-HSA-212165 | Epigenetic regulation of gene expression | 2/3 | 149/10704 | 0.001 | 0.002 | DNMT1/EZH2 | 2 |
| R-HSA-4655427 | SUMOylation of DNA methylation proteins | 1/3 | 17/10704 | 0.005 | 0.012 | DNMT1 | 1 |

## **
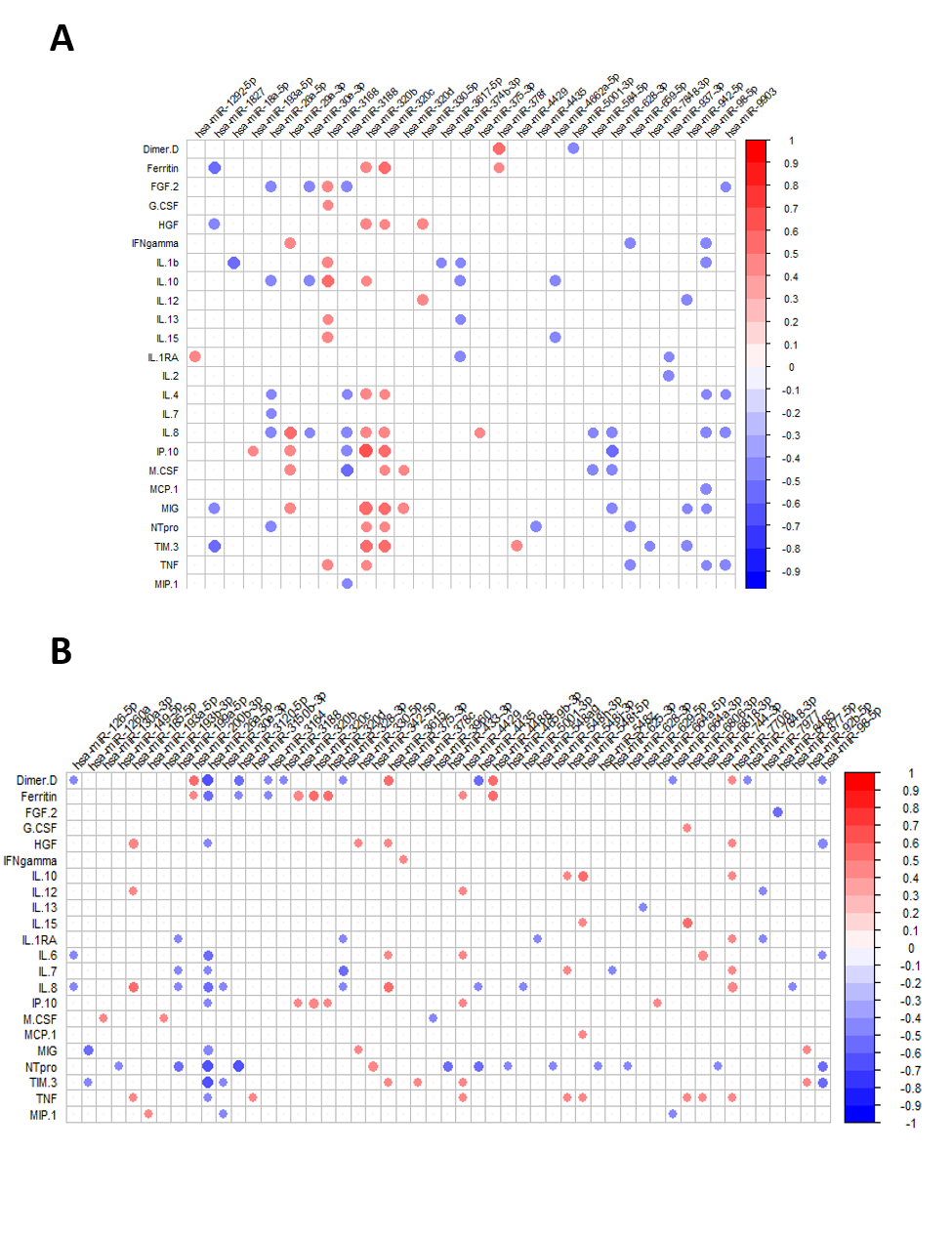
Supplementary Data 17**: Pearson correlation plot showing the correlation between SDE miRNAs and plasma cytokines / chemokines for: A) Moderate and B) Severe patients. The size of the circles is proportional to the strength of the correlation and the colour represents the direction (colour legends is shown on the right), where large dark blue represents a strong negative correlation and a large dark red circle represents a strong positive correlation. SDE miRNAs are on the horizontal axis and cytokines / chemokines on the vertical axis.

## **Supplementary Data 18: Clinical, epidemiological, and virological characteristics of SARS-CoV-2 infected patients stratified by mortality.**

|  | **Alive** | **Dead** | **p-value** |
| --- | --- | --- | --- |
| No. | 83 | 13 |  |
| Age (years) | 59 (49.2 - 69.4) | 83.7 (75.6 - 87) | **<0.001** |
| Gender (male) | 44 / 83 (53%) | 9 / 13 (69.2%) | 0.427 |
| Ethnicity (n=93) |  |  |  |
| *Caucasian* | 55 / 80 (68.8%) | 11 / 13 (84.6%) | 0.401 |
| *Hispanic* | 23 / 80 (28.8%) | 2 / 11 (15.4%) | 0.502 |
| BMI (kg/m^2^) (n=40) | 28.9 (26.1 - 34.5) | 23.5 (22.9 - 25.9) | 0.079 |
| *Smoker status* |  |  |  |
| *Smoker* | 8 / 83 (9.6%) | 0 / 13 (0%) | 0.452 |
| *Ex-smoker* | 13 / 83 (15.7%) | 3 / 13 (23.1%) | 0.790 |
| *Comorbidities* |  |  |  |
| Arterial hypertension | 33 / 83 (39.8%) | 9 / 13 (69.2%) | 0.090 |
| Cardiopathy | 13 / 83 (15.7%) | 4 / 13 (30.8%) | 0.349 |
| Chronic pulmonary disease | 12 / 83 (14.5%) | 4 / 13 (30.8%) | 0.286 |
| Chronic renal disease | 8 / 83 (9.6%) | 4 / 13 (30.8%) | 0.090 |
| Chronic liver disease | 2 / 83 (2.4%) | 0 / 13 (0%) | 0.999 |
| Chronic neurological disease | 10 / 83 (12%) | 4 / 13 (30.8%) | 0.175 |
| Neoplasia | 6 / 83 ( 7.2%) | 2 / 13 (15.4%) | 0.653 |
| Obesity | 20 / 83 (24.1%) | 0 / 13 (0%) | 0.105 |
| Diabetes | 19 / 83 (22.9%) | 1 / 13 (7.7%) | 0.375 |
| Chronic inflammatory disease | 3 / 83 (3.6%) | 1 / 13 (7.7%) | 0.999 |
| Autoinmune diseases | 4 / 83 (4.8%) | 0 / 13 (0%) | 0.950 |
| *Therapy* |  |  |  |
| *Basal* |  |  |  |
| NSAIDs | 2 / 83 (2.4%) | 2 / 13 (15.4%) | 0.153 |
| ACE inhibitors | 13 / 83 (15.7%) | 3 / 13 (23.1%) | 0.790 |
| ARA II | 7 / 83 (8.4%) | 2 / 13 (15.4%) | 0.774 |
| Corticoids | 9 / 83 (10.8%) | 1/ 13 (7.7%) | 0.999 |
| HIV antiretroviral therapy | 2 / 83 (2.4%) | 0 / 13 (0%) | 0.999 |
| *Treatment* |  |  |  |
| Chloroquine and hidroxychloroquine | 62 / 83 (74.7%) | 12 / 13 ( 92.3%) | 0.294 |
| Tocilizumab | 19 / 83 (22.9%) | 4 / 13 (30.8%) | 0.788 |
| Corticoids | 39 / 83 (47%) | 9 / 13 (69.2%) | 0.233 |
| COVID-19 related symptoms |  |  |  |
| Dyspnoea | 51 / 83 (61.4%) | 11 / 13 (84.6%) | 0.189 |
| Cough | 60 / 83 (72.3%) | 5 / 13 (38.5%) | **0.035** |
| Headache | 28 / 83 (33.7%) | 1 / 13 (7.7%) | 0.115 |
| Diarrhea or abdominal pain | 38 / 83 (45.8%) | 4 / 13 (30.8%) | 0.475 |
| Hospitalization |  |  |  |
| Hospital stay (days) (n=86) | 10 (7 - 20) | 11 (10 - 17) | 0.480 |
| Maximum temperature (n=85) | 38 (37.3 - 38.5) | 38.1 (37.45 - 38.8) | 0.540 |
| Oxygenotherapy | 50 / 83 (60.2%) | 13 / 13 (100%) | **0.013** |
| Invasive mechanical ventilation | 8 / 83 (9.6%) | 1 / 13 (7.7%) | 0.999 |
| Non- Invasive mechanical ventilation | 12 / 83 (14.5%) | 6 / 13 (46.2%) | **0.019** |
| Infiltrates | 66 / 83 (79.5%) | 13 / 13 (100%) | 0.159 |
| ICU | 12 / 83 (14.5%) | 1 / 13 (7.7%) | 0.820 |
|  |  |  |  |
| Exitus | 0 / 83 (0%) | 13 / 13 (100%) | **<0.001** |

## **Supplementary Data 19: miRNA mortality Risk Score (mRNA-MRS) results.**

Cox regression model is commonly used to study the relationship between predictor variables and survival time. In combination with least absolute shrinkage and selection operator (LASSO) method, it further allows both the variable selection and regularization to improve the prediction accuracy.

Here, all the 767 miRNAs were used as input for a LASSO Cox regression model using the glmnet R package (v4.1-3). For this, miRNA count data was first normalized using the variance-stabilizing transformation (VST), thus obtaining a matrix of homoscedastic values. A 10-fold cross-validation was used to select the optimal regression model. Specifically, we selected the most regularized model with a cross-validated error within one standard error of the minimum. This regression model identified 12 miRNAs with predictive ability: hsa-let-7f-1-3p, hsa-let-7g-5p, hsa-miR-1255a, hsa-miR-140-3p, hsa-miR-20a-5p, hsa-miR-22-3p, hsa-miR-3180-3p, hsa-miR-3180, hsa-miR-363-5p, hsa-miR-4510, hsa-miR-548h-3p and hsa-miR-6130. All of the selected miRNAs met the proportional hazards assumption with the exception of hsa-miR-548h-3p. This miRNA and hsa-miR-3180, which showed a very small regression coefficient (< 10^−15^), were removed from further analyses. Among the remaining 10 miRNAs, eight were associated with a higher probability of survival at 90 days and two miRNAs were associated with a worse prognosis (**Supplementary Data 20**).

This 10 miRNA-expression signature was used to generate a miRNA mortality risk score (miRNA-MRS) for each of the patients, based on their expression levels and Cox regression coefficients. The risk score formula was as follows: Risk score = - 0.198 ∗ Expr (hsa-let-7f-1-3p) - 0.153 ∗ Expr (hsa-let-7g-5p) - 0.111 ∗ Expr (hsa-miR-1255a) – 0.058 ∗ Expr (hsa-miR-140-3p) - 0.104 ∗ Expr (hsa-miR-20a-5p) + 0.304 ∗ Expr (hsa-miR-22-3p) + 0.106 ∗ Expr (hsa-miR-3180-3p) - 0.325 ∗ Expr (hsa-miR-363-5p) - 0.123 ∗ Expr (hsa-miR-4510) - 0.041 ∗ Expr (hsa-miR-6130).

The median value of the risk score was -3.992, which was used as a threshold to stratify the study subjects into low-risk (risk score < -3.992. n = 42) and high-risk (risk score ≥ -3.992. n = 42) score groups (**Figure 4A**). Kaplan-Meier analysis confirmed the significant differences in the overall survival (OS) (p-value <0.001) (**Figure 4B**). Moreover. ROC curve analysis confirmed the prognostic power of the miRNA-based risk score. The analysis of the 10-miRNA signature based model adjusted for age and gender showed a significantly higher AUC value than the model including only age and gender (0.968 vs. 0.881. p-value = 0.042), indicative of a higher sensitivity and specificity in predicting mortality of COVID-19 patients (**Figure 4C**). These results were confirmed by the multivariate Cox-regression model, highlighting the independent prognostic value of the risk-score (hazard ratio (HR) = 4.599; 95% CI (1.977− 10.700). p-value <0.001) (**Figure 4D**).

## **Supplementary Data 20: Mortality analysis with LASSO Cox regression model.** A. LASSO coefficient paths of all the miRNAs. Larger values of lambda regularization parameter shrink regression coefficients towards zero. B. Selection of optimal lambda parameter in the LASSO model. A vertical solid line indicates the selected lambda value based on 10-fold cross-validation (logarithmic scale). One standard error rule was applied to favour a parsimonious model.


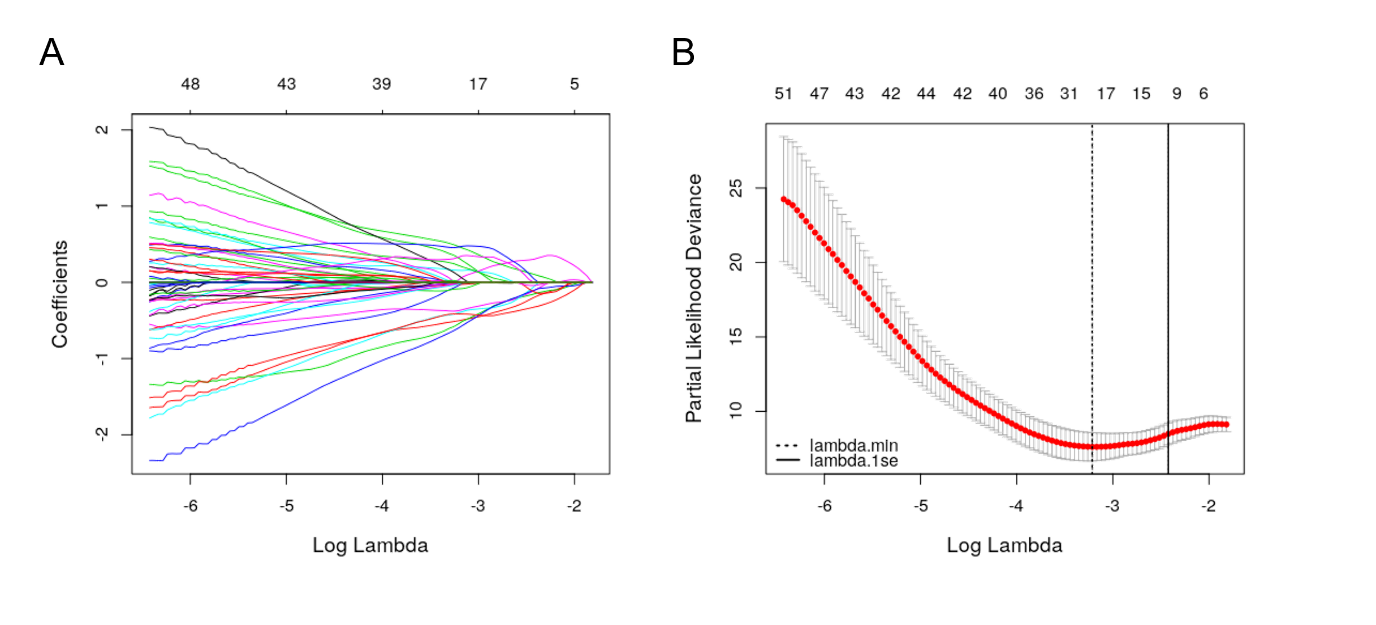


## **Supplementary Data 21: Forest plot displaying the 10 miRNAs included in the final miRNA Risk Score model.** The HR of each miRNA is indicated with 95% CI and statistical significance (p-value), LASSO least absolute shrinkage and selection operator; HR. hazard ratio; CI, confidence interval.

**
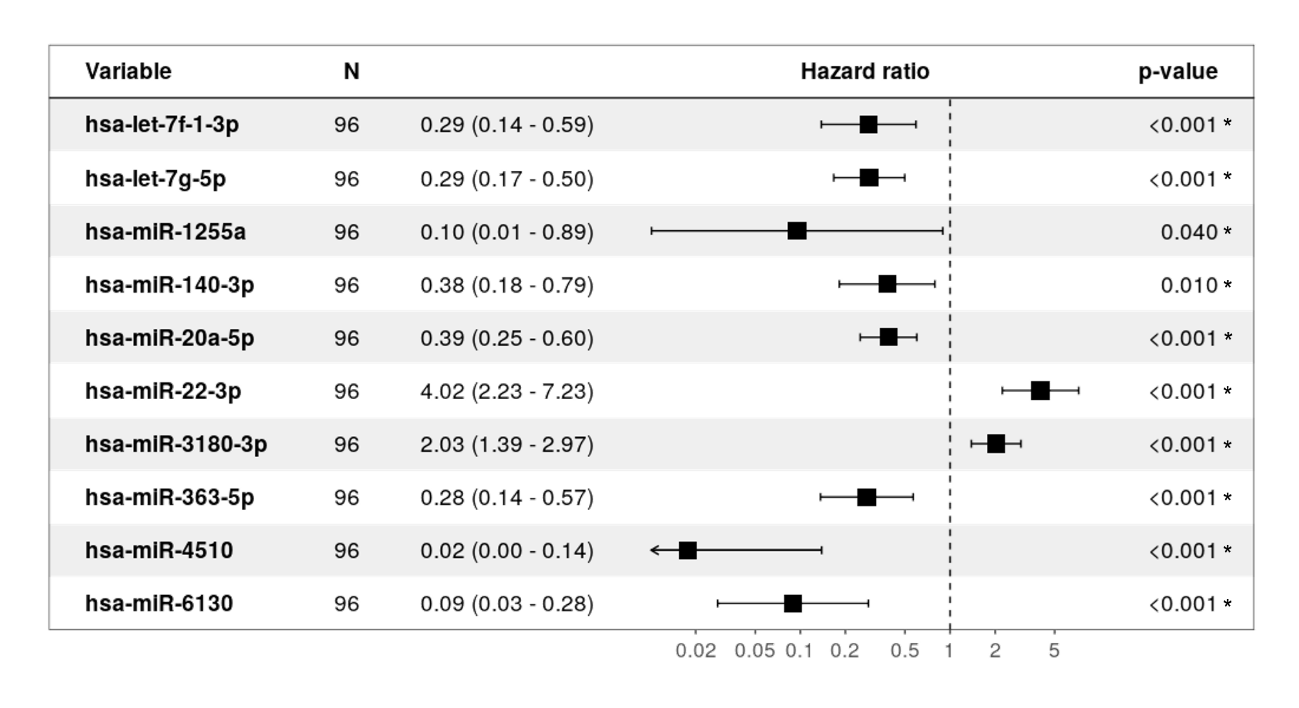
**

## **Supplementay Data 22.** Diagnostic accuracy of the model including the miRNA-MRS in combination with age and gender for predicting COVID-19-related death at 90 days.

| Variables | **Cut-off** | **TP** | **FP** | **TN** | **FN** | **Se** | **Sp** | **PPV** | **NPV** |
| --- | --- | --- | --- | --- | --- | --- | --- | --- | --- |
| Age + Gender | >1.205 | 10 | 12 | 71 | 3 | 76.9% | 85.5% | 45.5% | 95.9% |
| Age + Gender + miRNA-MRS | >0.670 | 12 | 6 | 77 | 1 | 92.3% | 92.8% | 66.7% | 98.7% |

Cut-off: Maximum value of sensitivity plus specificity; **Abbreviations**: FN. false negative; FP. false positive; TN. true negative; TP. true positive; NPV, negative predictive value; PPV, positive predictive value; Se. sensitivity; Sp. specificity.

**REFERENCES**

1. Andrews S. FastQC—A Quality Control Tool for High Throughput Sequence Data. 2016.

2. Martin M. **Cutadapt Removes Adapter Sequences From High-Throughput Sequencing Reads**. **EMBnetjournal**. 2011;17(1):10-2.

3. Kozomara A. Griffiths-Jones S. miRBase: annotating high confidence microRNAs using deep sequencing data. Nucleic Acids Res. 2014;42(Database issue):D68-73.
